# Preparation of levulinic acid and 5-(chloromethyl)furfural directly from terrestrial and marine biomass in a batch reactor under conventional and microwave heating

**DOI:** 10.1039/d6ra06834k

**Published:** 2026-09-02

**Authors:** Diona Avitha Aranha, Abhishek Kumar Yadav, Balamurugan Ayyakkalai, Vijayalekshmi Vijayakumar, Fillip Kumar Sarkar, Hemanth Giri Rao Vantharam Venkata, Sri Sailaja Nori, Saikat Dutta

**Affiliations:** a Department of Chemistry, National Institute of Technology Karnataka (NITK) Surathkal Mangalore 575025 Karnataka India sdutta@nitk.edu.in; b Sea6 Energy Pvt. Ltd, 1st Floor, C-CAMP, NCBS-TIFR, GKVK Campus Bellary Road Bengaluru 560065 Karnataka India

## Abstract

Lignocellulosic waste and seaweed represent second- and third-generation biomass resources, respectively, with significant potential as renewable feedstocks for the sustainable production of liquid fuels and value-added organic chemicals. In this study, the production of levulinic acid (LA) and 5-(chloromethyl)furfural (CMF), two important carbohydrate-derived platform chemicals, was investigated using selected lignocellulosic wastes and dried seaweed flakes (DSF) of *E. spinosum*. CMF was synthesized in a biphasic system comprising aqueous HCl (35%) and 1,2-dichloroethane, whereas LA was produced in 20% aqueous HCl under batch reaction conditions. The synthesis of both CMF and LA was examined under conventional heating and microwave irradiation (MWI). Furthermore, the effects of acid concentration, additives, reaction temperature, reaction time, catalyst loading, and biomass loading were systematically optimized to maximize product yields. Under the optimized conditions (20% aqueous HCl, 120 °C, 4 h), LA was obtained in 11 wt% yield, while MWI enabled an enhanced LA yield of 29 wt% from *E. spinosum* DSF within 30 min. CMF was produced in 13.5 wt% yield under optimized conditions (80 °C, 2 h). Selected terrestrial lignocellulosic wastes also furnished appreciable yields of CMF and LA under the optimized reaction conditions.

## Abstract 2

Lignocellulosic waste and seaweed represent second- and third-generation biomass resources, respectively, with significant potential as renewable feedstocks for the sustainable production of liquid fuels and value-added organic chemicals.

## Graphical abstract

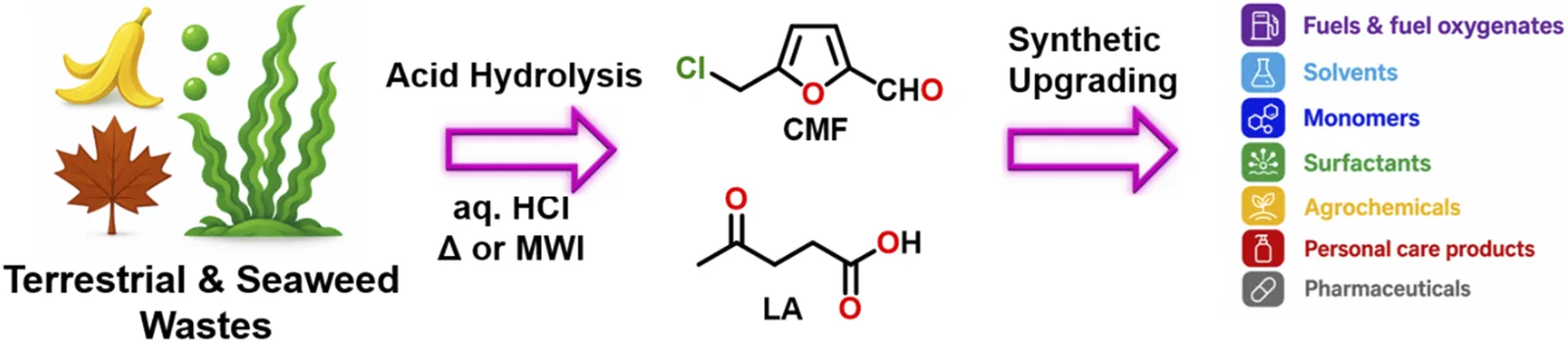

## 1 Introduction

Complex carbohydrates present in terrestrial lignocellulosic wastes constitute an abundant, commercially amenable, and environmentally sustainable source of renewable carbon to produce liquid transportation fuels and value-added organic chemicals.^1^ Such a strategy would satisfy multiple objectives, including effective waste mitigation, revenue generation, reduced anthropogenic carbon emissions, and the promotion of the local economy.^2^ However, the finite availability of terrestrial resources has intensified global interest in marine biomass as an alternative feedstock in the context of a blue economy.^3^ In this context, seaweeds (macroalgae) have emerged as promising third-generation biomass resources owing to their wide global distribution, rapid growth rates, abundant availability, and compatibility with advanced cultivation technologies.^4^ Unlike terrestrial biomass, seaweed cultivation does not compete with food production or require fertile land, fertilizers, pesticides, or freshwater irrigation, thereby offering distinct environmental and economic advantages.^5^

The conversion of terrestrial and marine biomass into specialty chemicals can be done following a two-step strategy. In the first step, complex polymeric carbohydrates are deconstructed into a limited number of reactive low-molecular-weight intermediates known as platform chemicals.^6^ In the second step, the inherent functionalities of these platform molecules are strategically exploited to synthesize structurally diverse organic compounds with tailored properties and applications. The conversion strategies of carbohydrates into platform chemicals can be broadly classified as thermochemical, chemocatalytic, or enzymatic processes.^7^ The chemical–catalytic processes are particularly interesting since they are fast, selective, catalytic in nature, energy-efficient, and reagent economic.^8^ Among the most important carbohydrate-derived platform chemicals are 5-(hydroxymethyl)furfural (HMF) and levulinic acid (LA), both primarily derived from hexose carbohydrates such as cellulose.^9,10^ LA can additionally be produced from pentose-rich hemicellulosic carbohydrates through alternative pathways. The conversion of cellulose into HMF involves hydrolytic depolymerization followed by acid-catalyzed intramolecular dehydration reactions.^11^ HMF retains all six carbon atoms of the parent sugar while partially reducing its oxygen content, thereby improving processability and chemical stability. Moreover, the transformation converts linear or cyclic sugar structures into a heteroaromatic (*i.e.*, furan) moiety. LA, a C5 platform chemical, is subsequently formed from HMF through acid-catalyzed hydration and ring-opening rearrangement reactions, with the concomitant formation of formic acid (FA) in equimolar quantities.^12^ The formation of the HMF intermediate was experimentally proven by our recent study and it is well-known in the literature.^13^ LA may also be produced from pentose sugars through furfuryl alcohol (FAL) intermediates.^14^

Both HMF and LA possess multiple reactive functionalities that enable their transformation into a broad range of commercially relevant derivatives.^15,16^ HMF contains hydroxymethyl, aldehyde, and furan functionalities, whereas LA possesses ketone, carboxylic acid, α-methyl, and α-methylene groups. These functionalities can be selectively manipulated individually or cooperatively to generate fuels, polymers, solvents, pharmaceuticals, and specialty chemicals.

Although HMF has been known in the literature for more than 150 years, its significance within carbohydrate-centric biorefineries has gained renewed attention over the past three decades.^17^ Extensive efforts have been devoted toward optimizing catalysts, reaction media, and heating methods for HMF production from first- and second-generation biomass resources.^18^ More recently, marine carbohydrates have also been explored as substrates for HMF synthesis.^19^ Despite these advances, the large-scale commercial production of HMF remains challenging, primarily due to its poor stability (hydrolytic, thermal, storage) and hydrophilic nature, complicating its separation from aqueous/polar reaction systems.^20^ Consequently, hydrophobic derivatives of HMF have attracted considerable attention due to their enhanced stability and easier product recovery *via* solvent extraction.^21^

5-(Chloromethyl)furfural (CMF) has emerged as one of the most promising hydrophobic analogs of HMF that can be synthesized directly from sugars, polymeric carbohydrates, and even untreated lignocellulosic biomass.^22^ Mechanistically, CMF is generated through HMF as a transient intermediate, where the hydroxymethyl group of HMF undergoes protonation followed by nucleophilic substitution with chloride ions.^23^ The reaction is typically performed in concentrated aqueous hydrochloric acid within an aqueous–organic biphasic system.^24^ Upon formation, CMF is continuously extracted into the organic phase, thereby minimizing hydrolysis back to HMF and suppressing subsequent degradation reactions.^25^ Hydrochloric acid offers several practical advantages, including low cost, bulk availability, and strong Brønsted acidity, making CMF an attractive renewable chemical intermediate for downstream value addition.^26^ The derivative chemistry of CMF has expanded substantially in recent years, demonstrating its practicality in accessing virtually all the derivatives of HMF and beyond.^27^

Parallel to the growing interest in CMF, LA has also emerged as a crucial carbohydrate-derived platform chemical.^10,16^ The derivative chemistry of CMF and LA is largely complementary; therefore, their selective synthesis under optimized conditions can substantially broaden the spectrum of renewable chemicals accessible from biomass-derived carbohydrates. We have previously reported the production of CMF and LA from both terrestrial (*e.g.*, cellulose) and marine carbohydrates (*e.g.*, chitin, carrageenan).^28,29^

The formation of humins, complex furanic polymeric materials enriched with oxygen-containing functionalities such as hydroxyl and carbonyl groups, is often unavoidable during HMF and LA synthesis.^30^ Humin is known by condensation reactions between sugar molecules, sugar-derived reactive intermediates (including HMF) formed during the dehydration steps, and other components of biomass.^31^ Owing to this challenge, most studies in the literature used purified carbohydrates to achieve higher selectivity and yields of CMF and LA. Consequently, the direct conversion of untreated biomass into HMF, CMF, and LA remains relatively underexplored.^32^

**Scheme 1:**
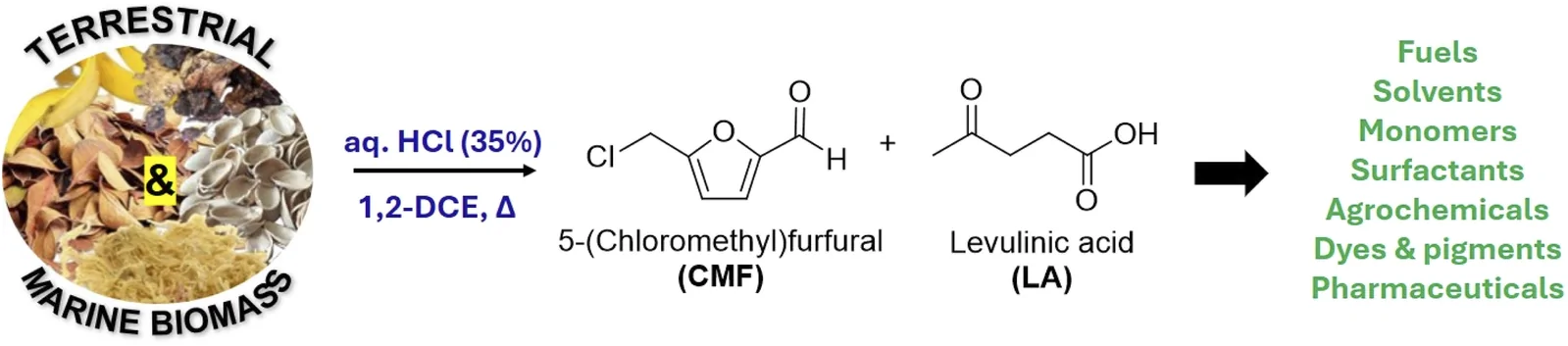
Production of CMF and LA as carbohydrate-derived platform chemicals from terrestrial and marine biomass for downstream synthetic value-addition.

Direct utilization of untreated or minimally processed biomass offers clear economic and environmental advantages by eliminating energy-intensive pretreatment and purification steps.^33^ The yield of CMF and LA from the real biomass depend on the degree of cellulose crystallinity, presence of other biopolymers (*e.g.*, lignin), and other inorganic and organic components present in them.^34^ The present work investigates the production of CMF and LA directly from selected terrestrial waste biomass, including forestry residues, food waste, and agro-industrial residues, which are widely available globally (Scheme 1). Sun-dried flakes of *E. spinosum* were selected as a representative marine biomass feedstock. *E. spinosum*, a red seaweed, is particularly rich in ι-carrageenan, a sulfated polymeric carbohydrate. Seaweeds are an excellent feedstock in a carbohydrate-centric biorefinery since they do not contain lignin, making their acid hydrolysis into platform chemicals relatively more straightforward and more selective under milder conditions.^35^ The synthesis of CMF and LA was carried out in batch reactors under both conventional heating and microwave irradiation (MWI) conditions. Based on our earlier studies, CMF production was performed in aqueous HCl (35%) using 1,2-dichloroethane (DCE) as the extraction solvent in a biphasic system, with intermittent product extraction intentionally avoided to simplify the process and improve scalability. LA production, on the other hand, was conducted in aqueous HCl (20%) at elevated temperatures. Furthermore, the water-insoluble humin by-product generated during the production of LA was isolated, characterized, and quantified to better understand biomass conversion pathways and carbon distribution during the process.

## 2 Experimental section

### 2.1 Materials

*Eucheuma denticulatum* (*spinosum*) seaweed was sourced from Bali, Indonesia, and the fresh seaweed was shredded into a slurry using a grinder. The juice and pulp from the seaweed were mechanically separated by squeezing, and the wet pulp was sun-dried to obtain *spinosum* Dried Seaweed Flakes (DSF), which was supplied by Sea6 Energy Pvt. Ltd (Bangalore, India). Silica gel (60–120 mesh), chloroform (99%), and ethyl acetate (99%) were procured from Loba Chemie Pvt. Ltd. Glacial acetic acid (99.5%) (LR grade) and aqueous hydrochloric acid (35%) were purchased from Molychem Pvt. Ltd. Anhydrous aluminium chloride (99%), *p*-toluenesulfonic acid (*p*TSA, 98%), anhydrous stannous chloride (97%), stannic chloride pentahydrate (98%), formic acid (98%), and chromium chloride (99%) were obtained from Spectrochem Pvt. Ltd. Amberlyst-15H, phosphotungstic acid (PTA, 99%), and Nafion NR50 were purchased from Sigma-Aldrich. Hexane was purchased from Hyma Synthesis Pvt. Ltd. The reactions were carried out in a 100 mL round-bottomed glass reactor equipped with a Teflon screw cap, procured from Sigma-Aldrich.

### 2.2 Instrumentations

The microwave-assisted syntheses were performed in an Anton Paar Monowave 200 microwave reactor. The ^1^H-NMR spectra were recorded in a Bruker NanoBay® NMR instrument operating at 400 MHz. The ^13^C-NMR spectra were recorded in the same instrument at a calculated operating frequency of 100 MHz. Fourier transform infrared (FTIR) spectra were acquired using the Attenuated Total Reflectance (ATR) method on a Bruker Alpha II FTIR spectrometer fitted with silicon carbide source. The sample was scanned 24 times with a scan rate of two scans per second and a spectral resolution of 4 cm^−1^ in the 600–4000 cm^−1^ spectral region. GEMINI 300 (Carl Zeiss, Germany) was used to record the Field-Emission Scanning Electron Microscopy (FESEM) images. EDAX PV 7600 SU system and Octane Super SDD detector (70 mm) was used to perform the energy-dispersive X-ray spectroscopy (EDX). The Brunauer–Emmett–Teller (BET) surface area measurements were done using an Autosorb IQ-XR-XR (Anton Paar, Austria). Powder X-ray diffraction (pXRD) analysis was performed using an Empyrean 3^rd^ Gen (Malvern PANalytical, Netherlands). TGA 4000 and a Spectrum 2 FT-IR spectrometer (PerkinElmer, Singapore) was used to perform the thermogravimetric analysis (TGA).

### 2.3 Preparation of LA from DSF by conventional heating

#### 2.3.1 Synthesis of levulinic acid (LA) from DSF

##### 2.3.1.1 Synthesis under conventional heating

*Spinosum*-dried seaweed flakes (DSF) (2.00 g) were charged in a round-bottom glass pressure reactor (100 mL) with a PTFE front-seal. 6 N HCl (20 mL) was added, and the reactor was sealed with a Teflon screw top. The reaction vessel was placed in a preheated oil bath at 120 °C and continuously stirred for 4 h. Upon completion of the reaction, the reactor was cooled to room temperature and diluted with 10 mL of distilled water. The insoluble humin was filtered by washing with 10 mL of distilled water. The aqueous layer was saturated with NaCl and cooled in ice-cooled water. Ethyl acetate (5 × 20 mL) was used to extract LA from aqueous solution. The organic layers were combined and dried over sodium sulfate. Ethyl acetate was removed under reduced pressure using a rotary evaporator, and LA was obtained as a brown liquid. The crude LA was then passed through a small plug of silica using ethyl acetate as a mobile phase to obtain pure LA as a light-yellow oil, with a yield of 0.216 g (11 wt%, 43 mol%). Alternatively, LA can be extracted by first distilling off aq. HCl through vacuum and then extracting it with ethyl acetate to remove the soluble humin, followed by passing through a small column of silica to obtain pure LA.

##### 2.3.1.2 Synthesis under MWI

A microwave G30 vial was charged with DSF (1.001 g) and aqueous HCl (20%, 10 mL), sealed with a rubber septum, and placed in a Monowave 200 microwave reactor. The reaction mixture was heated rapidly to 120 °C under continuous stirring at 1000 rpm, using the program to heat as soon as possible. At 120 °C, the hold time was set to 30 min at 1000 rpm. Afterwards, the reaction vial was cooled to 55 °C at 1000 rpm. Upon completion of the reaction, the mixture was cooled to room temperature and diluted with water (10 mL). LA was isolated and purified by the previously reported conventional heating method, affording 0.158 g (15.8 wt%, 62 mol%) of LA.

##### 2.3.1.3 Scaled-up synthesis of LA under reflux (conventional heating)

Dried seaweed flakes (DSF) of *E. spinosum* (70.0 g) and aqueous HCl (6 N, 700 mL) were introduced into a 1000 mL round-bottomed glass flask fitted with a water-cooled condenser and refluxed in a preheated oil bath (140 °C) for 5 h under continuous magnetic stirring. After the reaction, the reactor was cooled to room temperature under continued stirring, and the reaction mixture was filtered through a filter paper under vacuum to separate the black solid (insoluble humin). The insoluble humin collected on the filter paper was washed with deionized water (350 mL) to remove soluble humin. Then the obtained insoluble humin (black solid) was dried at 60 °C for 24 h (yield = 6.579 g). The filtrate was concentrated under reduced pressure using a rotary evaporator to obtain a mixture of soluble humin and LA. To the crude, 150 mL of deionized water was added, and LA was extracted with ethyl acetate (100 mL × 4). The organic layers were combined, dried over anhydrous Na_2_SO_4_, and evaporated under reduced pressure using a rotary evaporator to get the crude LA (7.589 g). LA was then purified using column chromatography (silica gel, 60–120 mesh) using ethyl acetate : hexane (1 : 1, v/v) as the eluent to afford a yield of 7.01 g (10 wt%, 39 mol%).

#### 2.3.2 Synthesis of 5-(chloromethyl)furfural (CMF) from DSF

##### 2.3.2.1 Synthesis under conventional heating

DSF (1.000 g), aq. HCl (35%, 20 mL) and DCE (30 mL) were charged in an ACE glass pressure reactor with a PTFE front-seal plug. The reactor was sealed and magnetically stirred at room temperature for 1 h, then heated in a pre-heated oil bath at 80 °C for 2 h. After completion of the reaction, the reactor was cooled down to room temperature and filtered using a Whatman grade 5 filter paper to remove the insoluble humin. The organic layer was separated and concentrated under reduced pressure to get CMF. The crude CMF was purified by column chromatography (Silica gel, 60–120 mesh) using chloroform as the eluent to obtain 0.135 g (13.5 wt%, 47 mol%) of CMF as a light-yellow oil.

##### 2.3.2.2 Synthesis under MWI

DSF (0.5 g), aq. HCl (35%, 4 mL) and DCE (6 mL) were added to a microwave G30 glass vial, which was sealed with a rubber septum. The vial was placed in a Monowave 200 microwave reactor and heated to 80 °C rapidly at 1000 rpm. At 80 °C, the hold time was set to 30 min at 1000 rpm. The reaction was subsequently cooled to 55 °C while continuously stirring at 1000 rpm. After completion of the reaction, the reaction mixture was cooled down to room temperature and filtered using a Whatman grade 5 filter paper to remove insoluble humin. CMF was extracted and purified according to the conventional heating protocol discussed earlier, yielding 0.06 g (12 wt%, 41.5 mol%) of CMF.

The moisture content of DSF of *E. spinosum* was found to be 16 wt% by drying the seaweed at 110 °C for 2 h. The molar yields of CMF and LA were calculated based on the dry seaweed weight and carbohydrate content of *E. spinosum*, which is assumed to be 70 wt% of the dry weight as reported in the literature.^36^ Thus, the mass of carbohydrates (ι-carrageenan) in 1.00 g of seaweeds was approximated to be 0.54 g. The following formulas can be used to calculate the yield of CMF and LA obtained from DSF.Yield (wt%) = [(mass of product obtained in gram)/(mass of biomass in gram)] × 100

Since 1 mol repeating unit of ι-carrageenan gives 2 moles of LA or CMF:Yield (mol%) = [(2 × moles of product obtained)/(moles of carbohydrate)] × 100

## 3 Results and discussion

### 3.1 Preparation of LA under conventional heating

The acid-catalyzed dehydration of *spinosum*-dried seaweed flakes (DSF) to LA was first studied under a batch-type reactor. DSF (2.0 g) and HCl (20 mL) were charged and placed in a preheated oil bath. After the completion of the reaction, the reaction mixture was filtered to remove the insoluble humin. The humin was washed with water to remove any acid residues, dried, and quantified. The crude LA obtained after extracting with ethyl acetate was passed through a small plug of silica to obtain pure LA.

**Fig. 1:**
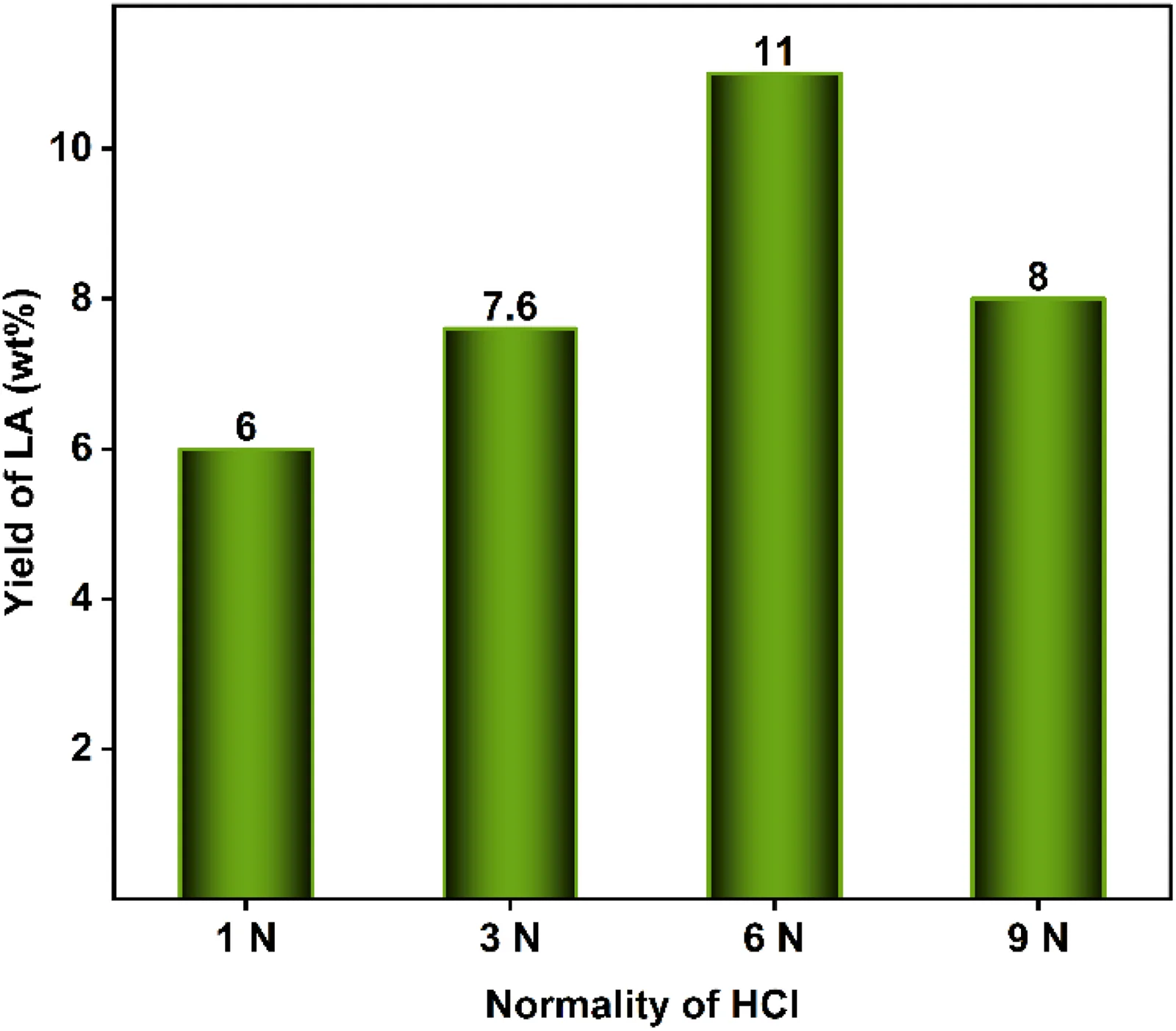
Effect of the acidic strength of HCl on the yield of LA. Reaction conditions: DSF (2.001 g), aq. HCl (20 mL), 120 °C, 4 h.

To begin our investigation, the transformation of DSF to LA was examined at varying HCl concentrations, with the reaction temperature maintained at 120 °C and the reaction time at 4 h. Acid hydrolysis of ι-carrageenan breaks the C–O–C glycosidic bonds, leading to the formation of galactose, which then undergoes a series of rearrangement steps to form the HMF intermediate. HMF then undergoes rehydration followed by ring-opening rearrangement into LA (Scheme S1, SI). When 1 N HCl was employed, LA was obtained in a relatively low yield (0.121 g, 6 wt%). Upon increasing the HCl concentration to 3 N, a slight increase in the LA yield (0.153 g, 7.6 wt%) was observed (Fig. 1). Further increase in acid concentration to 6 N led to a significant improvement in LA yield (0.216 g, 11 wt%). This is because when dilute HCl (1 N) is used, the dehydration process is slower, resulting in a lower yield. On the other hand, higher acid concentration indicates more protons in the reaction medium, hence favoring the dehydration of seaweed to LA. When the reaction was performed with 9 N HCl, the LA yield decreased, and more humin was observed. This can be attributed to side reactions and LA decomposition at higher acid conditions. Based on these results, 6 N HCl (20 mL) was found to be the optimal HCl concentration.

**Fig. 2:**
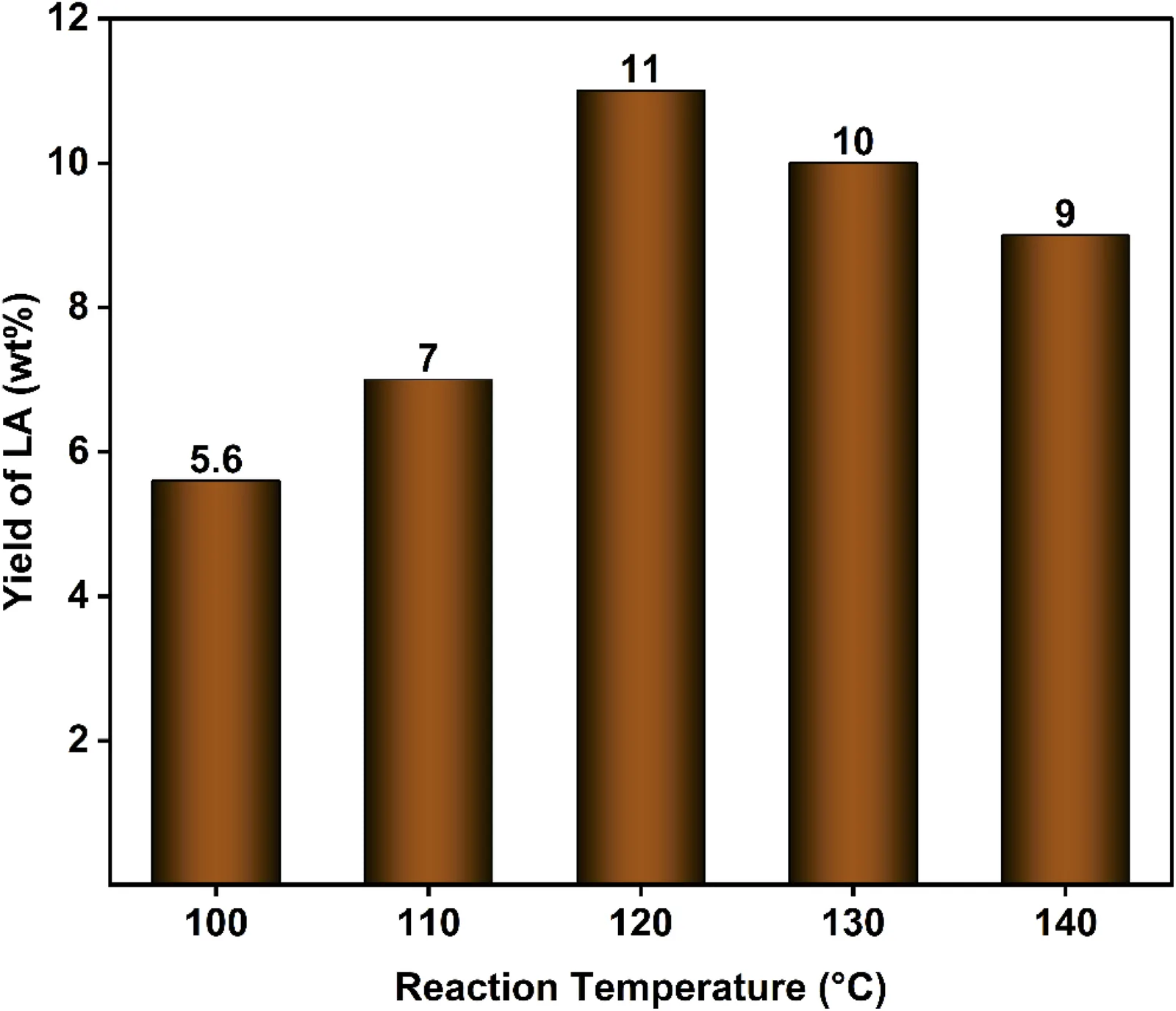
Effect of reaction temperature on LA yield. Reaction conditions: DSF (2.001 g), 20% aq. HCl (20 mL), 4 h.

After optimizing the acid concentration, the effect of temperature on LA yield was studied while keeping all other parameters constant. When the temperature was set to 100 °C, a sharp decrease in LA yield was observed, yielding 0.113 g (5.6 wt%) of LA. Afterwards, when the reaction was performed at 110 °C, the reaction didn't proceed at an appreciable rate and afforded a 0.144 g (7 wt%) yield of LA (Fig. 2). This can be justified by incomplete conversion due to insufficient thermal energy to promote the sequential hydrolysis, dehydration, and rehydration steps involved in LA formation. When the temperature was increased to 120 °C, the yield of LA increased to 0.216 g (11 wt%), attributed to the faster reaction rate. On further increasing the temperature to 130 °C and 140 °C, no appreciable change in the yield of LA was observed, and afforded 0.202 g (10 wt%) and 0.182 g (9 wt%) of LA, respectively. This decrease in LA yield at higher temperatures is due to partial decomposition of LA and increased formation of humin side products.

**Fig. 3:**
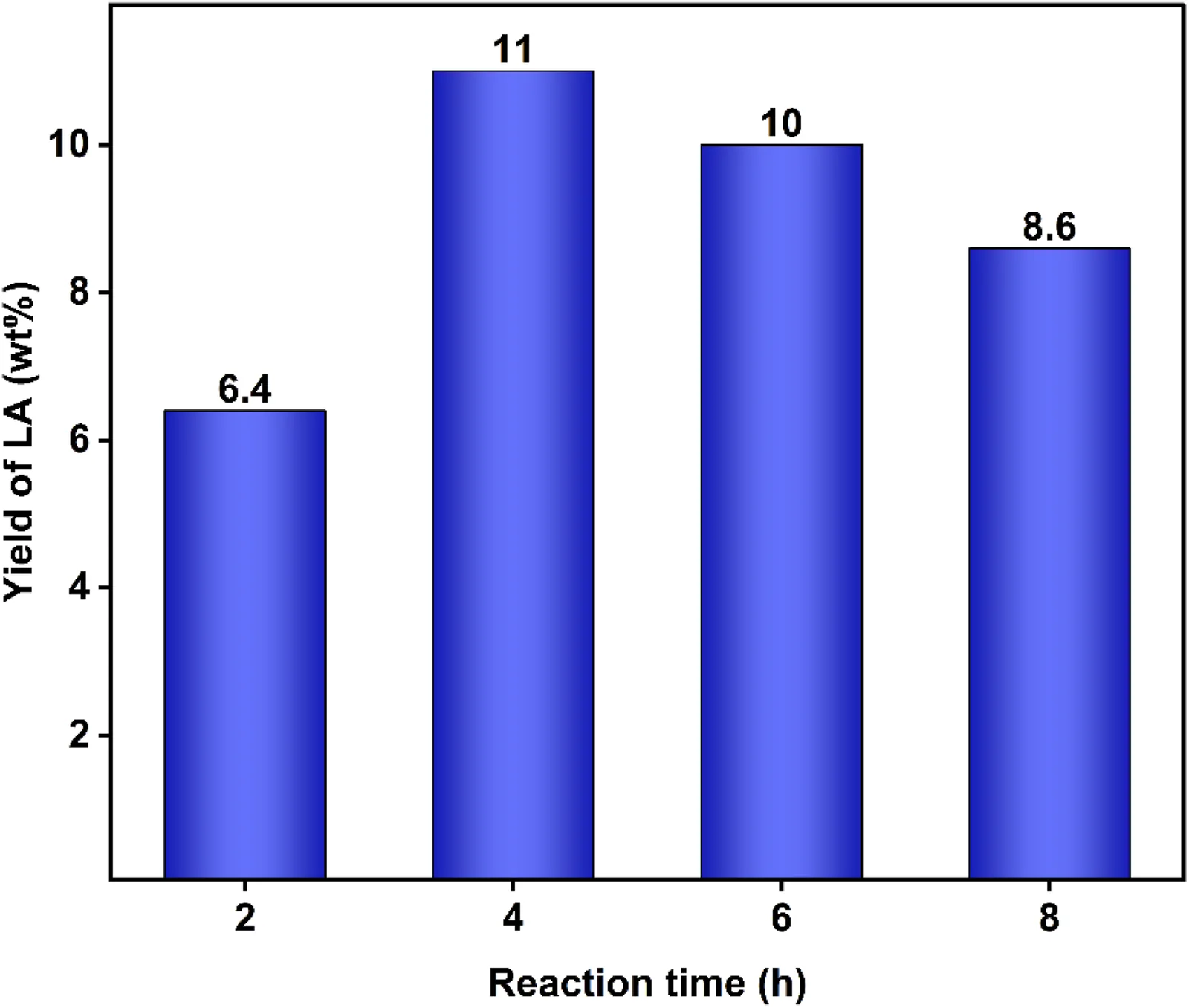
Effect of reaction duration on the yield of LA. Reaction conditions: DSF (2.001 g), 20% aq. HCl (20 mL), 120 °C.

Furthermore, the effect of reaction duration on LA yield was investigated. It was found that 4 h of reaction duration gave the maximum LA yield. Increasing the reaction time to 6 h and 8 h didn't yield a significant increase in yield, and 0.204 g (10 wt%) and 0.173 g (8.6 wt%) of LA were obtained, respectively. However, when the reaction was performed for 2 h at 120 °C, the yield of LA decreased to 0.129 g (6.4 wt%) (Fig. 3). To study the influence of acid strength and temperature on reaction kinetics and whether higher temperature can compensate for lower acid strength, 1 N HCl was used, although 6 N HCl gave a better yield. When the reaction was performed at 140 °C and 160 °C using 1 N HCl, 0.185 g (9 wt%) and 0.208 g (10.4 wt%) of LA were obtained, respectively. This observation suggests that under weak acidic conditions, increasing temperature can partially compensate for low acid strength.

When the batch-fed reaction was adopted, a significant increase in LA yield (0.253 g) was observed. In this approach, 0.5 g of DSF was added sequentially every hour for a total of 4 additions, rather than introducing the entire 2.00 g biomass at once. This controlled addition maintains a lower biomass concentration in the highly acidic medium, thereby reducing the rate of undesired condensation and polymerization reactions that lead to humin formation, while allowing more efficient conversion of freshly added DSF into LA.

**Fig. 4:**
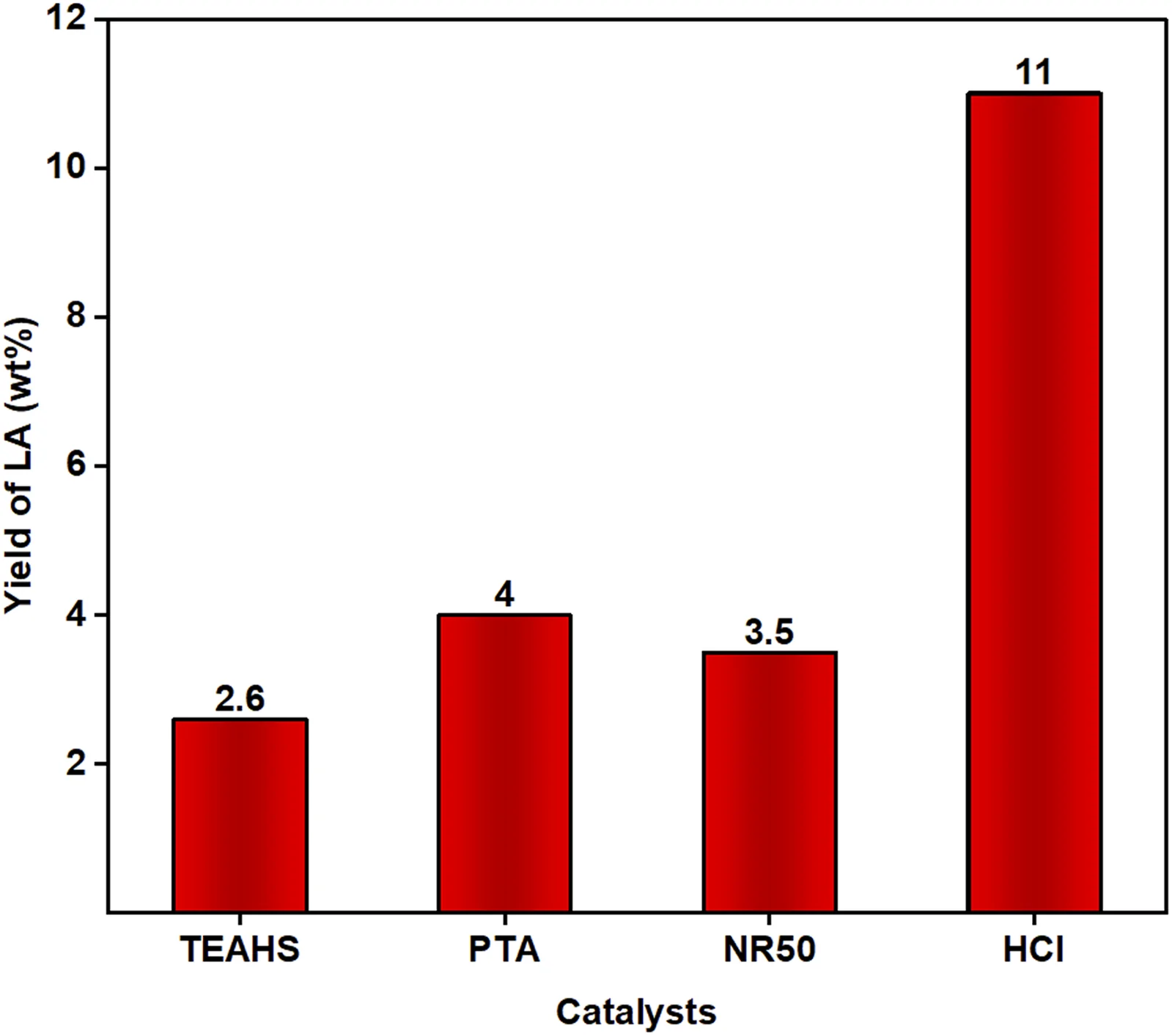
Effect of various catalysts on LA yield. Reaction conditions: DSF (2.001 g), 120 °C, 4 h.

Afterwards, the effect of various acid catalysts on LA yield was further studied under optimized conditions. When 2 g of triethylammonium hydrogen sulfate (TEAHS) and 20 mL of water were used, a sharp decrease in the yield of LA was recorded (0.052 g, 2.6 wt%). Increasing the catalyst loading of TEAHS to 2.5 g or the temperature to 140 °C did not yield any significant improvement in LA yield. Increasing the reaction time to 6 h didn't show a significant difference in LA yield. Under optimized conditions, with PTA (1.0 g) and water (20 mL), a sharp decrease in LA yield was observed, yielding 0.075 g (4 wt%) of LA. Using Nafion NR-50 (0.5 g) and water (20 mL) gave 0.071 g (3.5 wt%) of LA (Fig. 4). Elevated temperature and prolonged reaction time didn't show a drastic increase in LA yield.

**Fig. 5:**
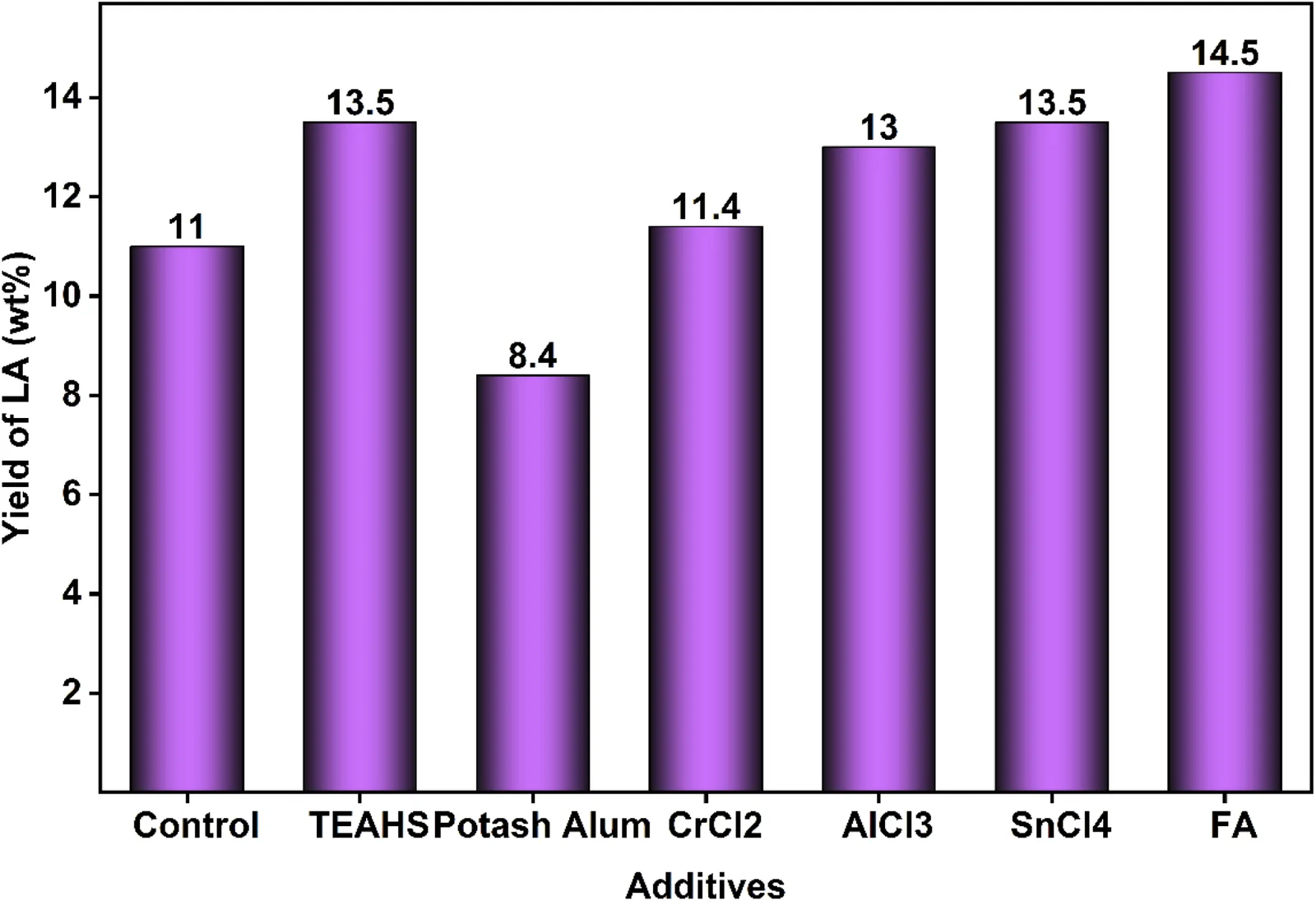
Effect of additives on LA yield. Reaction conditions: DSF (2.001 g), 20% aq. HCl (20 mL), 120 °C, 4 h.

Afterwards, the effect of various additives on LA yield was studied using 6 N HCl. The Lewis or Brønsted acidic additives help in the isomerization and dehydration steps. When a combination of 2 g of TEAHS and 6 N HCl (20 mL) was used as the catalyst, the LA yield increased significantly (0.271 g, 13.5 wt%) owing to the increased acidity of the reaction mixture. When 1 g of potash alum was used as an additive, the yield of LA decreased to 0.168 g (8.4 wt%). When metal halides, such as chromium chloride and aluminium chloride, were used as additives under optimized conditions, the LA yields were 0.229 g (11.4 wt%) and 0.259 g (13 wt%), respectively (Fig. 5). Similarly, when 1 g of stannic chloride was added to the catalyst under optimized conditions, the LA yield increased to 0.271 g (13.5 wt%). The synthesis of LA from DSF using formic acid as an additive was carried out following our previously reported method.^13^ A 0.29 g (14.5 wt%) LA containing 0.17 g of humin was obtained from 2.0 g of DSF.

Furthermore, to focus on the scalability of the synthesis of LA, a scaled-up (70 g) reaction was executed using 6 N HCl under reflux conditions at 140 °C for 5 h. Remarkably, the scale-up reaction proceeded smoothly to afford LA in 10 wt% yield, mirroring the efficiency observed in the smaller 2 g scale reaction. This scalability demonstrates its suitability for large-scale manufacturing and potential application in industry.

### 3.2 Preparation of LA under MWI

Microwave irradiation (MWI) has been shown to enhance selectivity toward furanic and LA during seaweed conversion. The rapid, uniform heating provided by MWI reduces thermal gradients, thereby facilitating the dehydration of carrageenan to HMF and its subsequent rehydration to LA. In the microwave-assisted synthesis of LA, DSF (1.0 g) was treated with 10 mL of 1.2 N HCl at 140 °C for 30 min. After completion, the insoluble humin was filtered using Whatman grade 5 filter paper, and LA was isolated using a procedure similar to conventional heating. At 140 °C, LA was obtained in 0.046 g (4.6 wt%) yield. Increasing the reaction temperature to 150 °C and 160 °C enhanced the reaction yield to 0.065 g and 0.089 g (9 wt%) of LA, respectively. Further increasing the temperature to 170 °C increased the yield of LA to 0.105 g (10.5 wt%). This enhancement can be attributed to the increased reaction kinetics. The elevated temperature promotes the breakdown of polymeric chains and accelerates the formation of HMF intermediates, thereby increasing LA production. However, further increase in temperature did not result in any significant change in LA yield. At higher temperatures, competing side reactions such as HMF polymerization and other condensation reactions may occur, leading to the formation of humin rather than additional LA.

Furthermore, the effect of acid concentration was examined using 6 N HCl. When the MW synthesis of LA was performed at 120 °C for 30 min, interestingly, the yield of LA increased to 0.158 g (15.8 wt%) with 0.07 g of humin. Afterwards, the reaction time was increased to 1 h, but no significant change in LA yield was observed.

### 3.3 Preparation of CMF under conventional heating

**Fig. 6:**
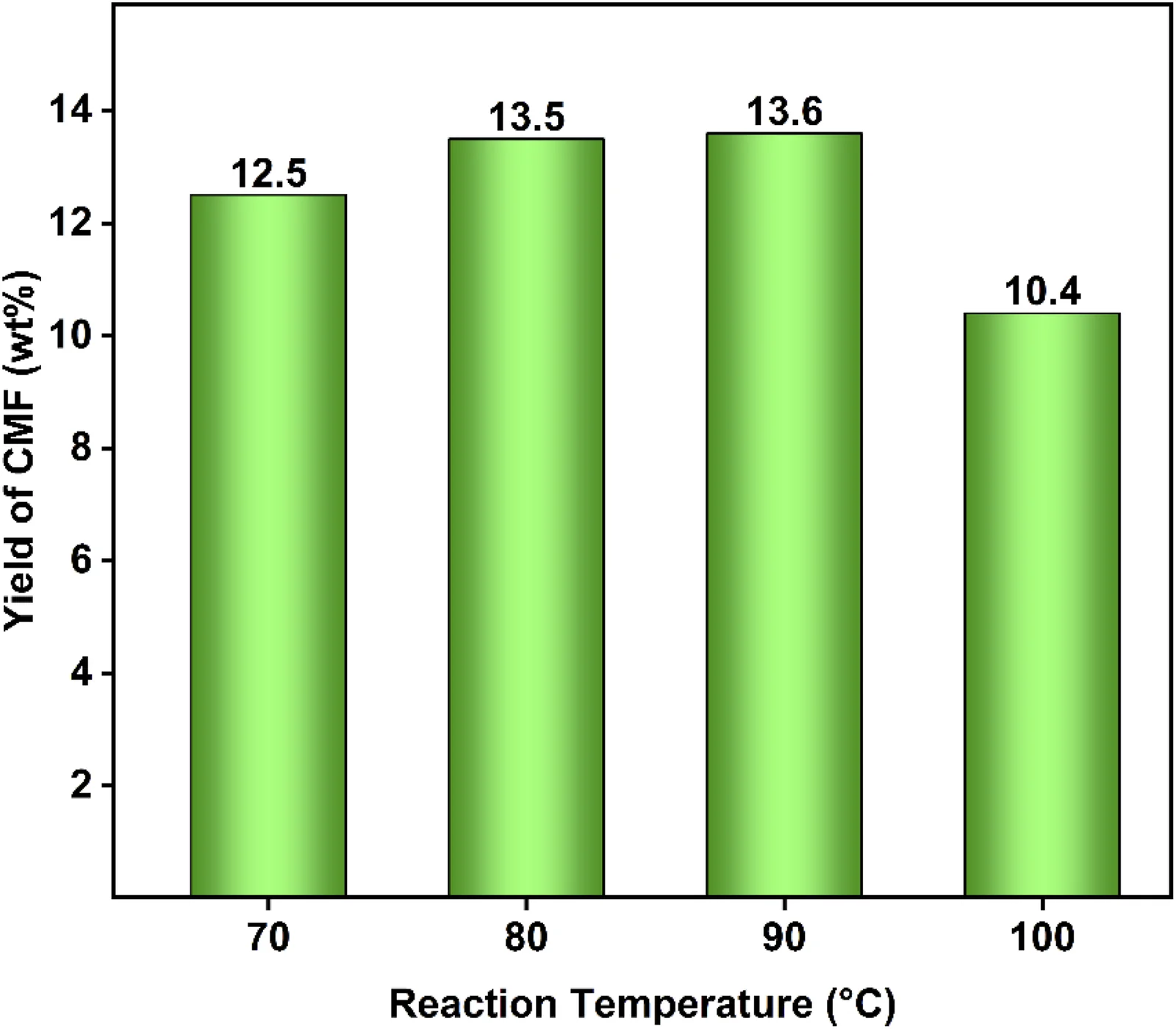
Effect of reaction temperature on the yield of CMF. Reaction conditions: DSF (1.001 g), 35% aq. HCl (20 mL), DCE (30 mL), 2 h.

The transformation of seaweed into CMF proceeds through multiple steps. Initially, the biomass (polysaccharide components present in the seaweed) undergoes hydrolysis, producing monosaccharides or smaller carbohydrate fragments. Further, the acid-catalyzed dehydration of these monomeric sugars gives platform chemical HMF. The strongly acidic conditions are suitable for polymeric chain breakdown of biomass into monomeric sugars and dehydration reactions. In the presence of hydrochloric acid, the HMF is further converted into CMF (Scheme S1). The reactions were performed in a round-bottomed glass pressure reactor equipped with a PTFE front-seal plug to maintain the required reaction conditions. Hydrochloric acid was selected as the catalyst due to its strong acidity, low cost, commercial availability, and easy recovery by simple distillation after the reaction. The synthesis of CMF was initially carried out under biphasic conditions employing aqueous HCl (aq. 35%) and 1,2-dichloroethane (DCE). The major challenge in selecting the appropriate extracting organic solvent for the CMF production is the presence of concentrated HCl. Most solvents that are considered to be green solvents (*e.g.*, ester, alcohol) are not suitable here due to their instability under the reaction conditions. Chlorinated solvents (*e.g.*, chloroform, DCE) are known for their effectiveness in extracting CMF from the aqueous acid. DCE is a commercial solvent available in bulk quantities, has a suitable boiling point, and is chemically stable.^26^ The biphasic reaction medium plays a pivotal role during this transformation. As soon as CMF is formed, it migrates into the DCE organic phase, which limits exposure to the aqueous acidic conditions and reduces the risk of hydrolyzing back to HMF and subsequent decomposition reactions into humin. Initially, the effect of reaction temperature on CMF formation was examined. When the reaction was conducted at 80 °C for 2 h using 20 mL of aqueous HCl (35%) and 30 mL of DCE, CMF was obtained in a yield of 0.135 g (13.5 wt%). Increasing the reaction temperature to 90 °C resulted in a modest increase in the CMF yield, affording 0.136 g (13.6 wt%) of CMF (Fig. 6). Further increasing the reaction temperature to 100 °C decreased the yield of CMF to 0.104 g (10.4 wt%), possibly due to CMF decomposition at higher temperatures.

**Fig. 7:**
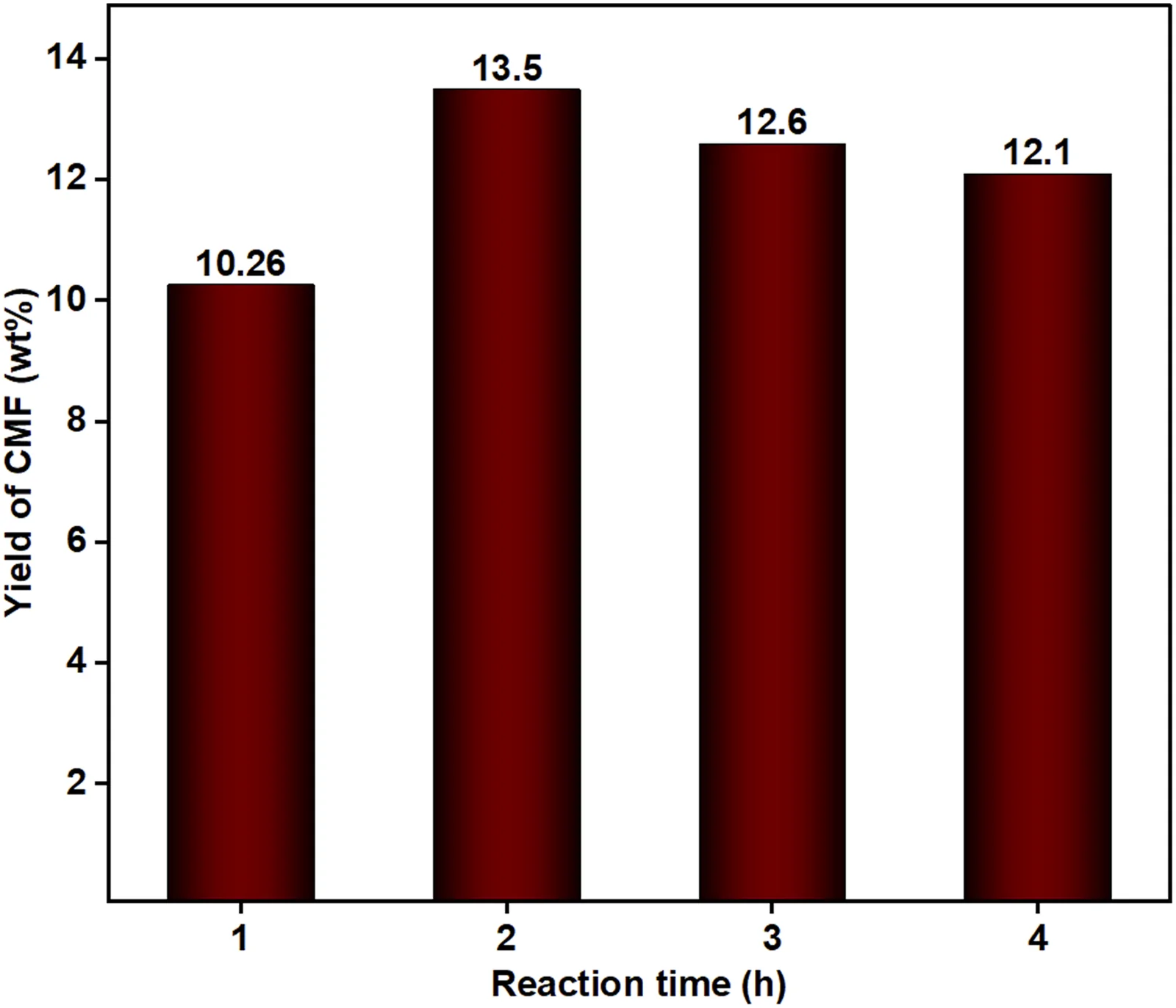
Effect of reaction duration on the yield of CMF. Reaction conditions: DSF (1.001 g), 35% aq. HCl (20 mL), DCE (30 mL), 80 °C.

After optimizing the reaction temperature, the effect of reaction time on CMF yield was examined while keeping the other parameters constant. Extending the reaction time to 3 h and 4 h decreased the yield of CMF, affording 0.126 g (12.6 wt%) and 0.121 g (12.1 wt%), respectively (Fig. 7). The decline in yield over extended reaction time suggests that the CMF can gradually degrade or undergo hydrolysis or participate in side reactions that lead to increased humin formation. Reducing the reaction time to 1 h also decreased the CMF yield, likely due to incomplete conversion of the polysaccharides in DSF. Overall, the results demonstrate that both reaction temperature and duration significantly affect the efficiency of CMF production from DSF. Under the reaction conditions investigated, a reaction temperature of 80 °C and a reaction time of 2 h provided the highest CMF yield.

To evaluate the broader applicability of the proposed method, the optimized reaction protocols were extended to other naturally occurring biomass wastes, including flower waste, banana peel, tender coconut shell, dry tree leaves, areca nut husk, pistachio shells, and de-oiled cashew nut shells. The successful formation of CMF and LA from these precursors indicates that the methodology is not just limited to seaweed but can also be adapted to diverse lignocellulosic and non-lignocellulosic feedstocks. All the waste biomass was cleaned, dried in a hot-air oven, ground, and sieved to get uniform size distributions. In addition to CMF yield, humin formation was quantified to better understand mass balance. Since humin forms from competing side reactions, its quantification provides insights into the efficiency of CMF formation.

Initially, the mixture of waste flowers was employed. The sieved flower waste (1.0 g), DCE (30 mL), and conc. HCl (20 mL) was charged in a 100 mL glass pressure reactor fitted with a Teflon screw top. The reactor was placed in a pre-heated oil bath set at 80 °C, and the reaction was performed for 2 h. A similar workup procedure to that used for the seaweed-derived biomass was followed. We obtained 17.5 wt% of pure CMF with 0.272 g of humin. Banana peels, which are rich in carbohydrates, were then investigated as a precursor. The CMF yield obtained was 25 wt% with 0.292 g of humin. Areca nut husk, characterized by its higher lignocellulosic content and rigid structure, showed a comparatively lower CMF yield of 13.2 wt% and 0.374 g of humin.^37^ The higher lignin content in arecanut husk promotes carbonization, thereby increasing the humin formation and decreasing the yield. Tender coconut biomass (after removal of the outer green layer) was also evaluated. Owing to its fibrous nature and moderate cellulose yield, we obtained a CMF yield of 24 wt% and 0.389 g of humin. CMF synthesis from cashew nut shells was also explored. The crude cashew nut shells were refluxed with toluene to remove the oil. The CMF yield obtained from de-oiled cashew nut shells was 12.6 wt%, and 0.215 g of humin was formed. Pista shells, representing a hard lignocellulosic biomass with high structural rigidity, were also evaluated for CMF synthesis. Due to their relatively high lignin content and dense matrix, a lower yield of CMF (12.8 wt%) compared to softer biomass precursors such as banana peels and flower residue was obtained, with a corresponding humin yield of 0.402 g. Dry tree leaves derived from *Syzygium samarangense* (water apple) were employed, which yielded 8.4 wt% of CMF with 0.480 g of humin. Similarly, microwave irradiation (MWI) experiments were performed, and the comparative results of conventional and microwave heating are tabulated in Table 1. The wt% yield of CMF and LA for all the biomass precursors in Tables 1 and 2 was calculated by using the following equation:Yield (wt%) = [(mass of product obtained in gram)/(mass of starting material in gram)] × 100

**Table 1:** Synthesizing CMF from various biomass precursors under conventional heating and MWI^a^

Entry	Biomass	Conventional synthesis		Microwave synthesis	
		Yield of CMF (wt%)	Insoluble humin (g)	Yield of CMF (wt%)	Insoluble humin (g)
1	Flower waste	17.5	0.272	14.8	0.155
2	Banana peels	25.0	0.292	12.0	0.185
3	Areca nut husk	13.2	0.374	11.8	0.262
4	Tender coconut shells	24.0	0.389	9.8	0.277
5	De-oiled cashew shells	12.6	0.215	7.6	0.200
6	Pistachio shells	12.8	0.402	9.2	0.185
7	Dried tree leaves	8.4	0.480	5.4	0.250

a Reaction conditions: conventional synthesis: biomass (1.0 g), HCl (20 mL), DCE (30 mL), 80 °C for 2 h. Microwave irradiation: biomass (0.5 g), HCl (6 mL), DCE (4 mL), 80 °C for 30 min.

**Table 2:** Synthesizing LA from various biomass precursors under conventional heating and MWI with FA^a^

Entry	Biomass	Conventional synthesis		Microwave synthesis	
		Yield of LA (wt%)	Insoluble humin (g)	Yield of LA (wt%)	Insoluble humin (g)
1	Flower waste	22.4	0.290	16.0	0.155
2	Banana peels	13.5	0.350	18.0	0.167
3	Areca nut husk	21.5	0.458	26.0	0.215
4	Tender coconut shells	25.2	0.437	24.4	0.221
5	De-oiled cashew shells	17.8	0.340	28.0	0.160
6	Pistachio shells	18.1	0.402	7.6	0.115
7	Dried tree leaves	15.0	0.580	19.6	0.290

a Reaction conditions: conventional synthesis: biomass (1.0 g), 20% aq. HCl (18 mL), FA (2 mL), 120 °C for 4 h. Microwave irradiation: biomass (0.5 g), 20% aq. HCl (9 mL), FA (1 mL), 120 °C for 1 h.

In addition to CMF synthesis, LA production from the same set of biomass precursors was investigated. A control experiment was initially performed using flower waste in the absence of formic acid under the standard reaction conditions (1.0 g feedstock, 20 mL of 20% aq. HCl, 120 °C, 4 h). A comparatively lower yield of LA (0.127 g, 12.7 wt%) with increased amount of insoluble humin (0.339 g). Therefore, formic acid was employed as additive for all biomass feedstocks for the improved production of CMF and LA. A comparative summary of LA and humin yields for various biomass precursors, obtained *via* conventional and microwave synthesis, is presented in Table 2.

### 3.4 Characterization of humin obtained from DSF of *E. spinosum*

The humin formed during LA synthesis was recovered by filtering the reaction mixture and washing it multiple times with water to remove acids and soluble humin. The humin was then dried in a hot-air oven and powdered for further analysis.

**Fig. 8:**
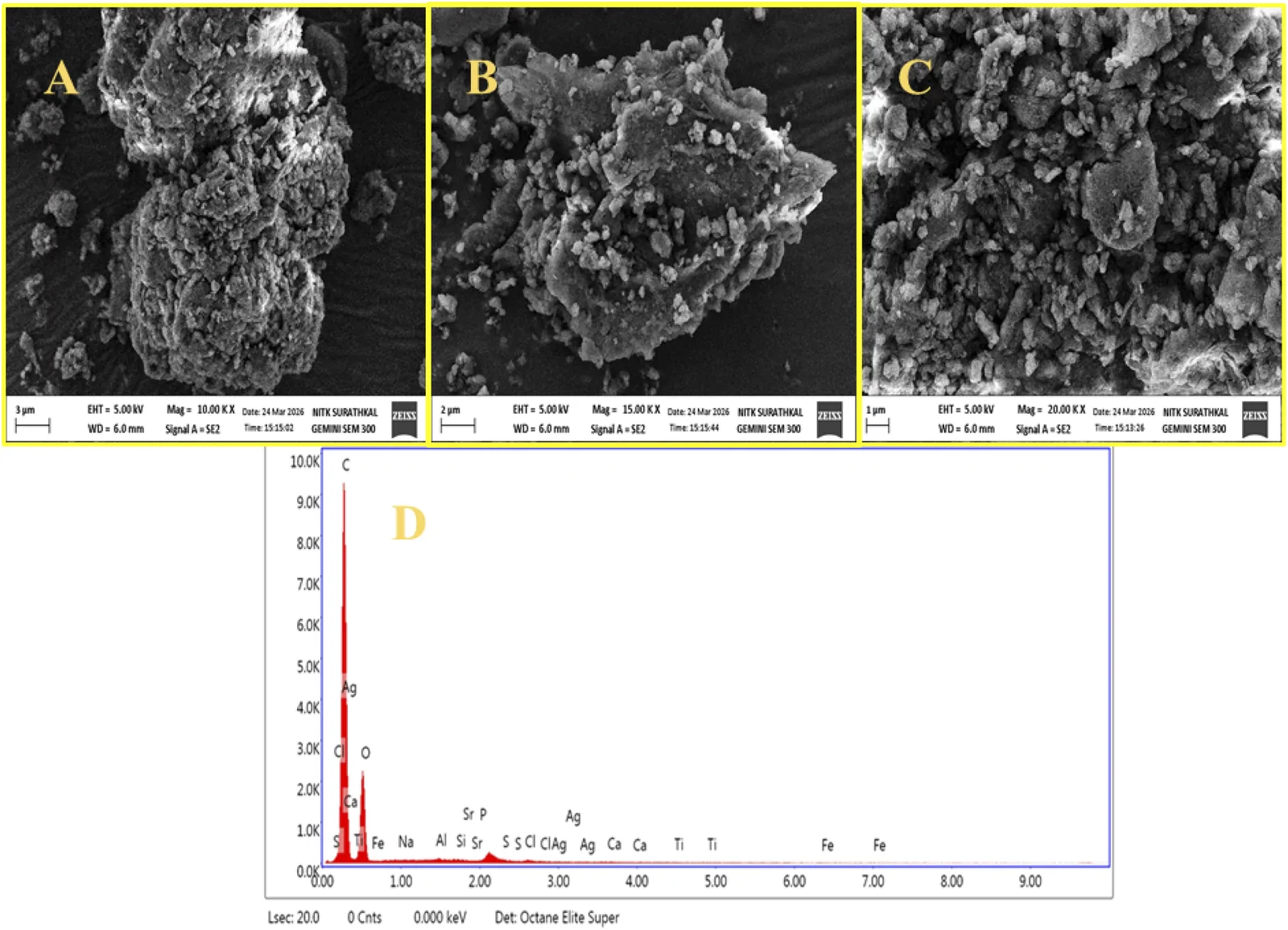
The FESEM images of insoluble humin produced during LA synthesis from DSF of *E. spinosum* at different magnifications (A–C) and the EDX spectrometry (D).

Morphological features and their elemental compositions of the humin were effectively investigated by SEM-EDX analysis (Fig. 8). The energy-dispersive X-ray spectra exhibited sharp peaks corresponding to the elements carbon (76.95%), oxygen (18.81%), sulfur (1.09%), and chlorine (0.71%).

**Fig. 9:**
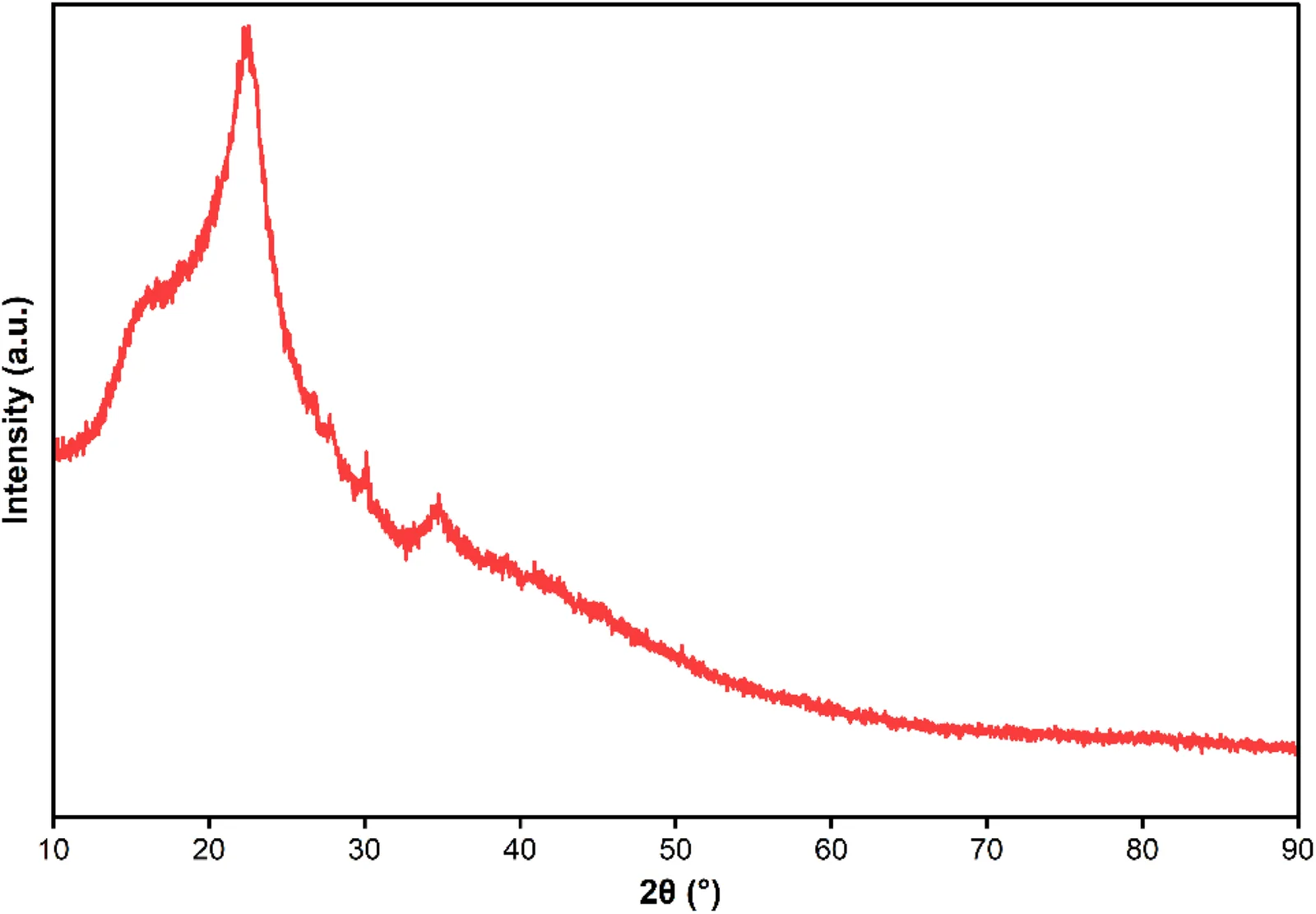
Powder XRD diffractogram of insoluble humin produced during LA synthesis from DSF of *E. spinosum*.

Powder X-ray diffraction spectroscopy was performed to confirm the amorphous nature of the insoluble humin. The broad diffraction peak around 2*θ* ≈ 22° in Fig. 9 confirms the amorphous nature.

**Fig. 10:**
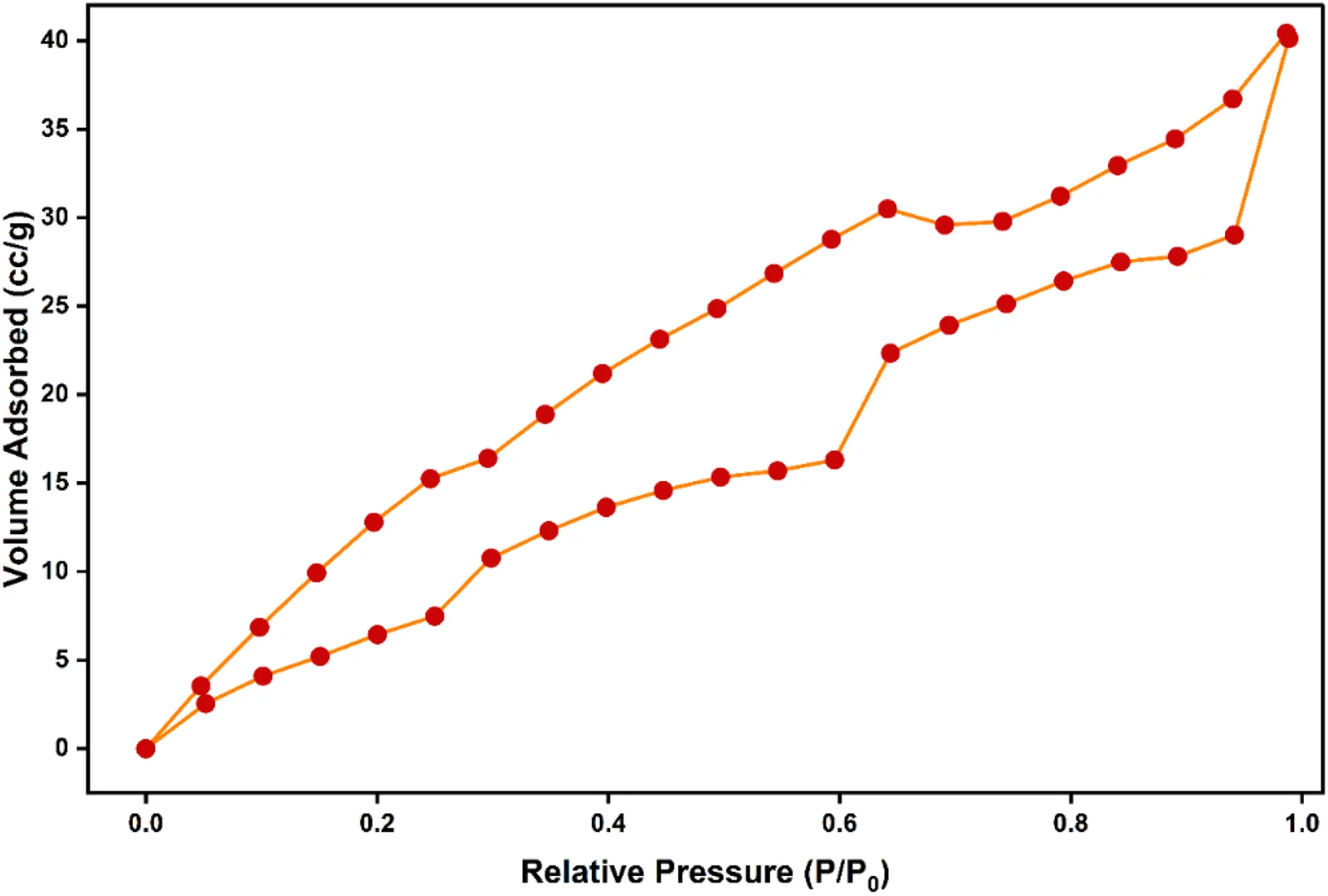
Nitrogen adsorption–desorption isotherm of humin produced during LA synthesis from DSF of *E. spinosum*.

The specific surface area, pore size distributions, and pore diameters of LA humin were analyzed using BET nitrogen adsorption and desorption isotherms, and the results are shown in Fig. 10. This is a type IV pattern with an H3-type hysteresis loop, indicating a very irregular pore structure. The specific surface area was found to be 22.960 m^2^ g^−1^. The total pore volume and average pore diameter were 0.062 cm^3^ g^−1^ and 5.92 nm, respectively. The average pore diameter revealed the presence of mesopores.

**Fig. 11:**
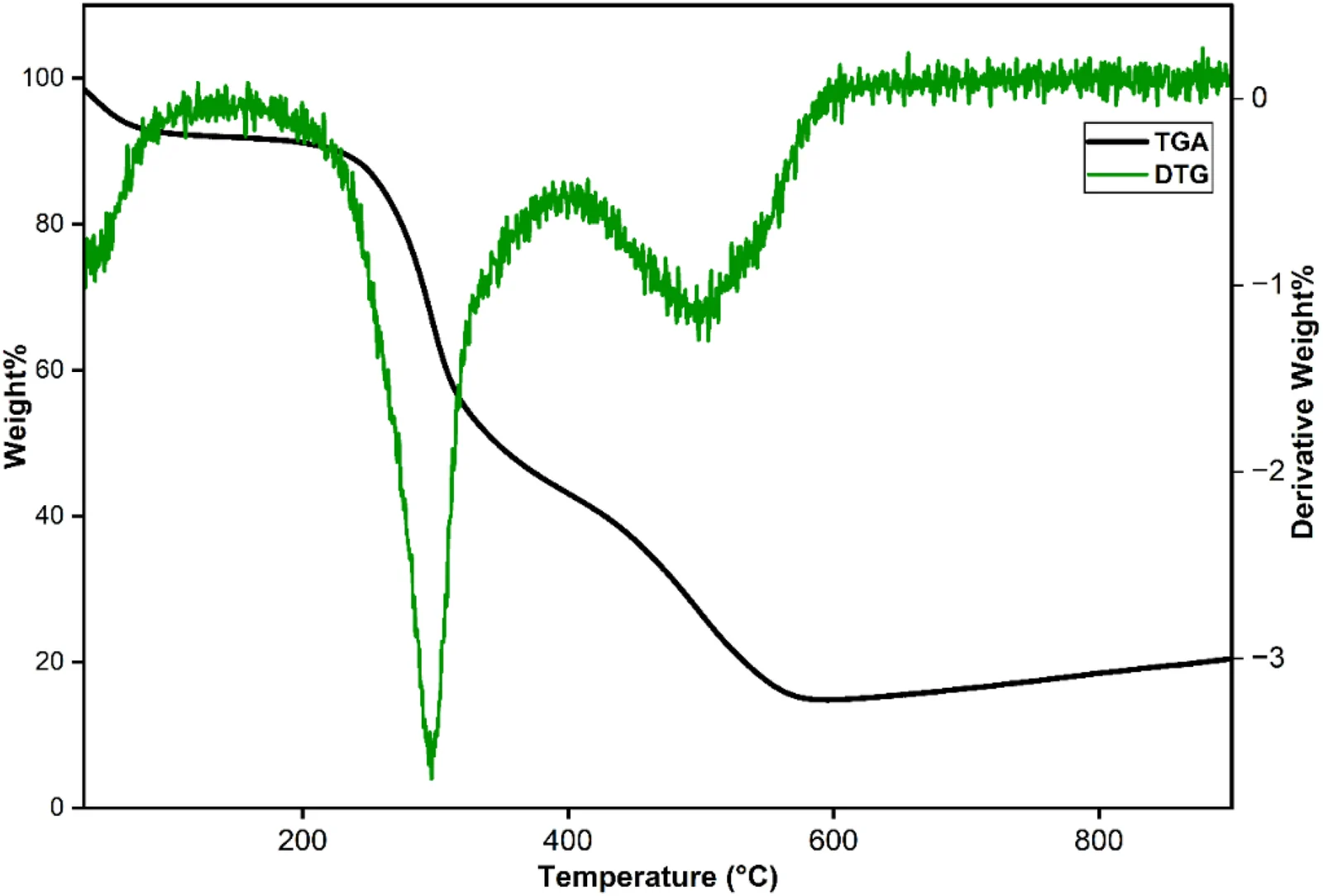
TGA and DTG indicate thermal degradation of humin produced during LA synthesis from DSF of *E. spinosum*.

The thermal stability of LA-derived humin was studied by thermogravimetric analysis (TGA) and differential thermogravimetric analysis (DTG), as shown in Fig. 11. The TGA data was consistent with the industrial humin reported in the literature.^30^ The first stage of decomposition around 100 °C was mainly due to evaporation of surface-bound moisture in humin. The largest weight loss, around 300 °C, is due to the decomposition of partially dehydrated carbohydrate residues. It can also be due to the degradation of oxygenated functional groups in the furanic resin *via* dehydration and decarboxylation reactions since the humin is rich in furanics and oxygenated functional groups.^38,39^ The sharp DTG curve around 300 °C suggests the loss of volatile organic compounds. A broader secondary degradation region between approximately 350 and 600 °C can be attributed to further deoxygenation and carbonization of the furanic resins.

The TGA and XRD patterns of the humin derived from DSF were comparable to those of the humin derived from banana peels, as reported in the literature.^40^ The minor differences observed in the TGA and XRD profiles can be attributed to lignin in terrestrial biomass, suggesting that humin from terrestrial biomass shows structural similarities with humin derived from seaweeds. Given its characteristics and properties, humin can be a sustainable source of carbon-based material for conversion into activated carbon.

## 4 Conclusion

This work demonstrates the efficient conversion of seaweed into LA and CMF through acid-catalyzed processes. The yield of LA was influenced by acid concentration, temperature, reaction duration, and the presence of additives. Optimal conditions were achieved using 6 N HCl at 120 °C for 4 h, while batch-fed feedstock addition effectively suppressed humin formation and improved the LA yield. Incorporation of Lewis acids alongside Brønsted acids further enhanced the LA yield. CMF was prepared in a HCl : DCE biphasic mixture. Moreover, microwave-assisted synthesis was also explored to produce CMF and LA. The developed methodology for the synthesis of CMF and LA was also extended to various terrestrial biomass precursors under both conventional and MWI.

## Author contributions

Diona Avitha Aranha and Abhishek Kumar Yadav worked on experiments, product purification, characterization, and process optimization. Sea6 Energy provided the seaweed sample, assisted in the process optimization, and edited the manuscript. SD conceptualized the work, supervised the progress, and wrote the original manuscript.

## Conflicts of interest

The authors declare no competing interests.

## Supplementary Material

RA-016-D6RA06834K-s001

## Data Availability

The data supporting this article have been included as part of the supplementary information (SI). Supplementary information: spectroscopic (FTIR, ^1^H-NMR, and ^13^C-NMR) characterization data of CMF and LA, photographs of the microwave reaction trials, temperature programming, and the process scale up studies. See DOI: https://doi.org/10.1039/d6ra06834k.

## References

[cit1] Dusselier M. , Mascal M. and Sels B. F., in Selective Catalysis for Renewable Feedstocks and Chemicals, ed. K. M. Nicholas, Springer International Publishing, Cham, 2014, vol. 353, pp. 1–40

[cit2] Leong H. Y., Chang C.-K., Khoo K. S., Chew K. W., Chia S. R., Lim J. W., Chang J.-S., Show P. L. (2021). Biotechnol. Biofuels.

[cit3] Saiyad M., Shah N., Joshipura M., Dwivedi A., Pillai S. (2026). ChemistrySelect.

[cit4] Cakmak E. K., Owusu-Agyeman I., Caglar Balkis N., Aksu A., Taskin O. S., Cetintasoglu M. E., Yenipazar H., Saygun A., Sahin Yesilcubuk N., Kocatürk-Schumacher N. P., Moges M. E., Morken J., Salazar S. A., Gomez d'Ayala G., Cerruti P., Muñana S., Ruiz Rubio L., Yuksel Imer D., Cagrican Gok E., Tunc I., Karahan B. D., Keles O., Bas B., Binello F., Daniotti S., Zeni A., Cetecioglu Z. (2026). Resour., Conserv. Recycl..

[cit5] Veríssimo N. V., Mussagy C. U., Oshiro A. A., Mendonça C. M. N., Santos-Ebinuma V. D. C., Pessoa A., Oliveira R. P. D. S., Pereira J. F. B. (2021). Green Chem..

[cit6] Mika L. T., Cséfalvay E., Németh Á. (2018). Chem. Rev..

[cit7] Kumari A. , Shrivastava S., Pandey A. and Taherzadeh M. J., Sustainable Technologies for Value Addition to Biomass Waste, Elsevier, 2026

[cit8] Dutta S. (2024). ACS Omega.

[cit9] Mittal A., Pilath H. M., Johnson D. K. (2020). Energy Fuels.

[cit10] Jeanmard L., Rongwong W., Chisti Y. (2025). Biomass Bioenergy.

[cit11] Huang K., Song J., Su K., Xu X., Jetti K. D., Xu Y., Zhou X., Kidder M. K., Smith J. C., Mohan M. (2026). npj Mater. Sustainability.

[cit12] Istasse T., Richel A. (2020). RSC Adv..

[cit13] Aranha D. A., Yadav A. K., Dutta S. (2026). ChemistrySelect.

[cit14] An S., Song D., Sun Y., Zhang Q., Zhang P., Guo Y. (2018). ACS Sustainable Chem. Eng..

[cit15] Xia H., Xu S., Hu H., An J., Li C. (2018). RSC Adv..

[cit16] Dutta S., Bhat N. S. (2021). ChemCatChem.

[cit17] Aranha D. J., Gogate P. R. (2023). Ind. Eng. Chem. Res..

[cit18] Arabi N., Babaei S., Qi J., Arvelli S., Zhao J. (2026). Biomass Futures.

[cit19] Wagh A. S., Ukarde T. M., Pandey P. H., Lali A. M., Pawar H. S. (2019). ACS Sustainable Chem.
Eng..

[cit20] Rosenfeld C., Konnerth J., Sailer-Kronlachner W., Solt P., Rosenau T., van Herwijnen H. W. G. (2020). ChemSusChem.

[cit21] Anchan H. N., Dutta S. (2023). Biomass Convers. Biorefin..

[cit22] Hong M., Guo Y., Chen S., Xie A., Zhu W., Han J., Liu S. (2024). Int. J. Biol. Macromol..

[cit23] Chen B., Li Z., Feng Y., Hao W., Sun Y., Tang X., Zeng X., Lin L. (2021). ChemSusChem.

[cit24] Mascal M., Nikitin E. B. (2010). Green Chem..

[cit25] Bueno Morón J., Arbore F., van Klink G. P. M., Mascal M., Gruter G. M. (2024). ChemSusChem.

[cit26] Mascal M. (2019). ACS Sustainable Chem. Eng..

[cit27] Karimi S., Gharouni Fattah S., Li Z., Zuo M., Nasrollahzadeh M., Zeng X. (2025). Green Chem..

[cit28] Onkarappa S. B., Dutta S. (2019). ChemistrySelect.

[cit29] Naik C P., Yadav A. K., Aranha D. A., Dutta S. (2025). Biomass Convers. Biorefin..

[cit30] Constant S., Lancefield C. S., Vogelzang W., Pazhavelikkakath Purushothaman R. K., Frissen A. E., Houben K., de Peinder P., Baldus M., Weckhuysen B. M., van Es D. S., Bruijnincx P. C. A. (2024). Green Chem..

[cit31] de Jong E., Mascal M., Constant S., Claessen T., Tosi P., Mija A. (2025). Green Chem..

[cit32] Menegazzo F., Ghedini E., Signoretto M. (2018). Molecules.

[cit33] Cheah W. Y., Sankaran R., Show P. L., Ibrahim T. N. B. T., Chew K. W., Culaba A., Chang J.-S. (2020). Biofuel Res. J..

[cit34] Jakob A., Likozar B., Grilc M. (2025). ChemSusChem.

[cit35] Trivedi N., Reddy C. R. K., Radulovich R., Jha B. (2015). Algal Res..

[cit36] Tong H. K., Lee K. H., Wong H. A. (1979). Int. J. Food Sci. Technol..

[cit37] Julie Chandra C. S., George N., Narayanankutty S. K. (2016). Carbohydr. Polym..

[cit38] Árvai C., Medgyesi Z., Lui M. Y., Mika L. T. (2024). Adv. Synth. Catal..

[cit39] Velasco Calderón J. C., Arora J. S., Mushrif S. H. (2022). ACS Omega.

[cit40] Al-sareji O. J., Grmasha R. A., Meiczinger M., Al-Juboori R. A., Somogyi V., Hashim K. S. (2024). Materials.

