# Supplementary material for: Preparation of levulinic acid and 5-(chloromethyl)furfural directly from terrestrial and marine biomass in a batch reactor under conventional and microwave heating
Source: RSC Adv. 2026 Sep 2;16(42):51731–41. doi: 10.1039/d6ra06834k (PMC13537382; doi:10.1039/d6ra06834k)
Supplement: Supplementary file 1 [file RA-016-D6RA06834K-s001.pdf]

Supplementary Information (SI) for RSC Advances.
This journal is © The Royal Society of Chemistry 2026

Supplementary Information

Preparation of levulinic acid and 5-(chloromethyl)furfural directly from terrestrial and marine biomasses in a batch reactor under conventional and microwave heating

*Diona Avitha Aranha,[1†] Abhishek Kumar Yadav,[1†] Balamurugan Ayyakkalai,[2] Vijayalekshmi Vijayakumar,[2] Fillip Kumar Sarkar,[2] Hemanth Giri Rao Vantharam Venkata,[2] Sri Sailaja Nori[2] and Saikat Dutta[1]**

[1] Department of Chemistry, National Institute of Technology Karnataka (NITK), Surathkal, Mangalore-575025, Karnataka, India.

[2] Sea6 Energy Pvt. Ltd., 1st Floor, C-CAMP, NCBS-TIFR, GKVK Campus, Bellary Road, Bengaluru-560065, Karnataka, India.

[†] Equal contribution

* Corresponding author. E-mail: sdutta@nitk.edu.in

Number of Pages: 09

Number of Figures: 10

Number of Tables: 01

Number of Schemes: 01

Characterization data of synthesized compounds

5-(Chloromethyl)furfural (CMF): ^{1}H-NMR ($CDCl_3$, 400 MHz, δ ppm): 4.50 (s, 2H), 6.49 (d, 1H, J = 3.6 Hz), 7.11 (d, 1H, J = 3.6 Hz), 9.47 (s, 1H). ^{13}C-NMR ($CDCl_3$, 100 MHz, δ ppm): 36.5, 112.1, 122.3, 152.7, 155.9, 177.6; FTIR (cm^{-1}): 2848, 1674, 1518, 1262, 1022.

Levulinic acid (LA): ^{1}H-NMR (D_2O, 400 MHz, δ ppm): 2.07 (s, 3H), 2.43 (t, 2H, J = 6.4 Hz), 2.70 (t, 2H, J = 6.4 Hz). ^{13}C-NMR (D_2O, 100 MHz, δ ppm): 27.6, 28.9, 37.5, 176.8, 213.1; FTIR (cm^{-1}): 3576, 2922, 1711, 1369, 1165.

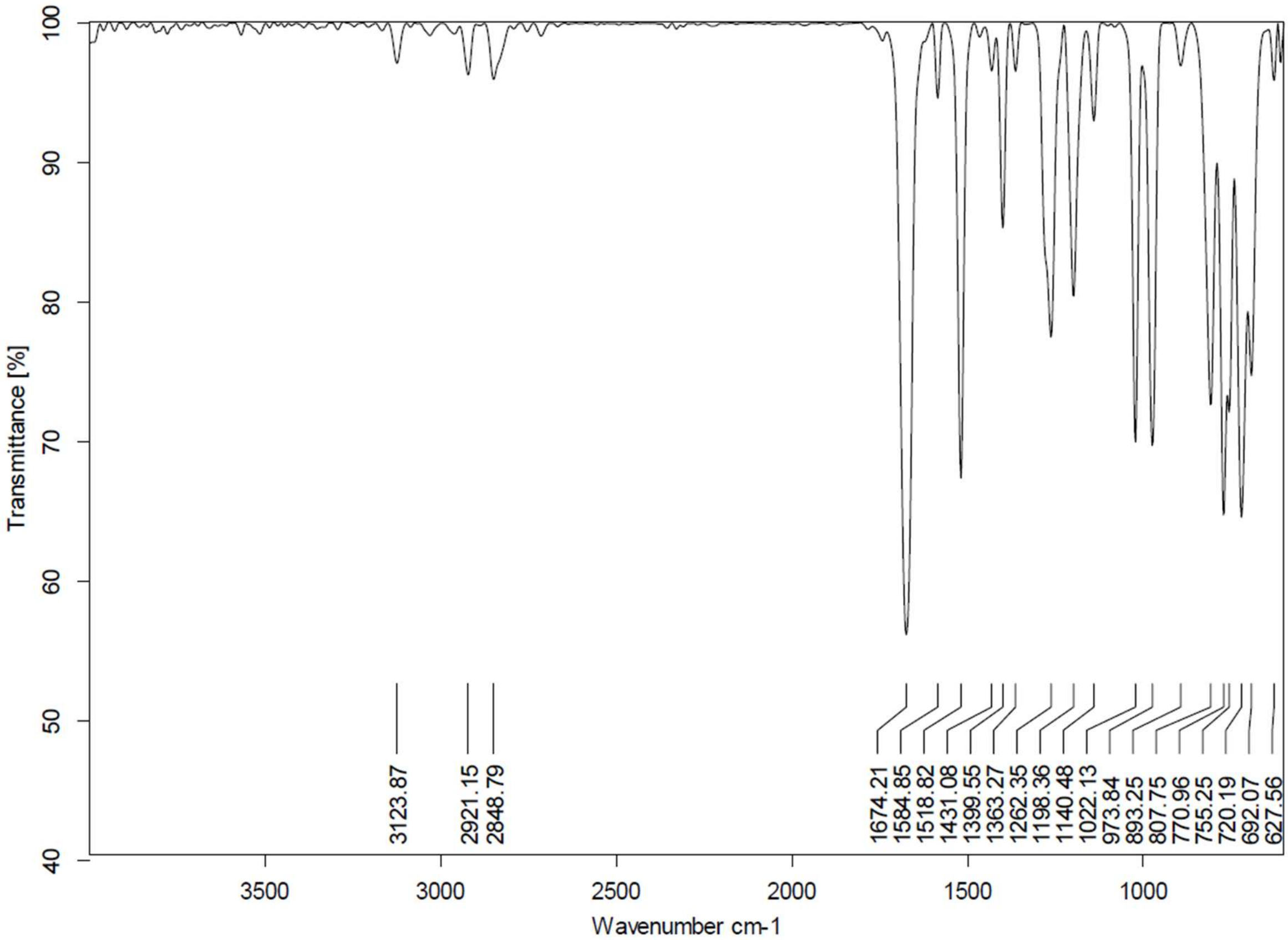

Figure S1. The FTIR spectrum of **CMF**.

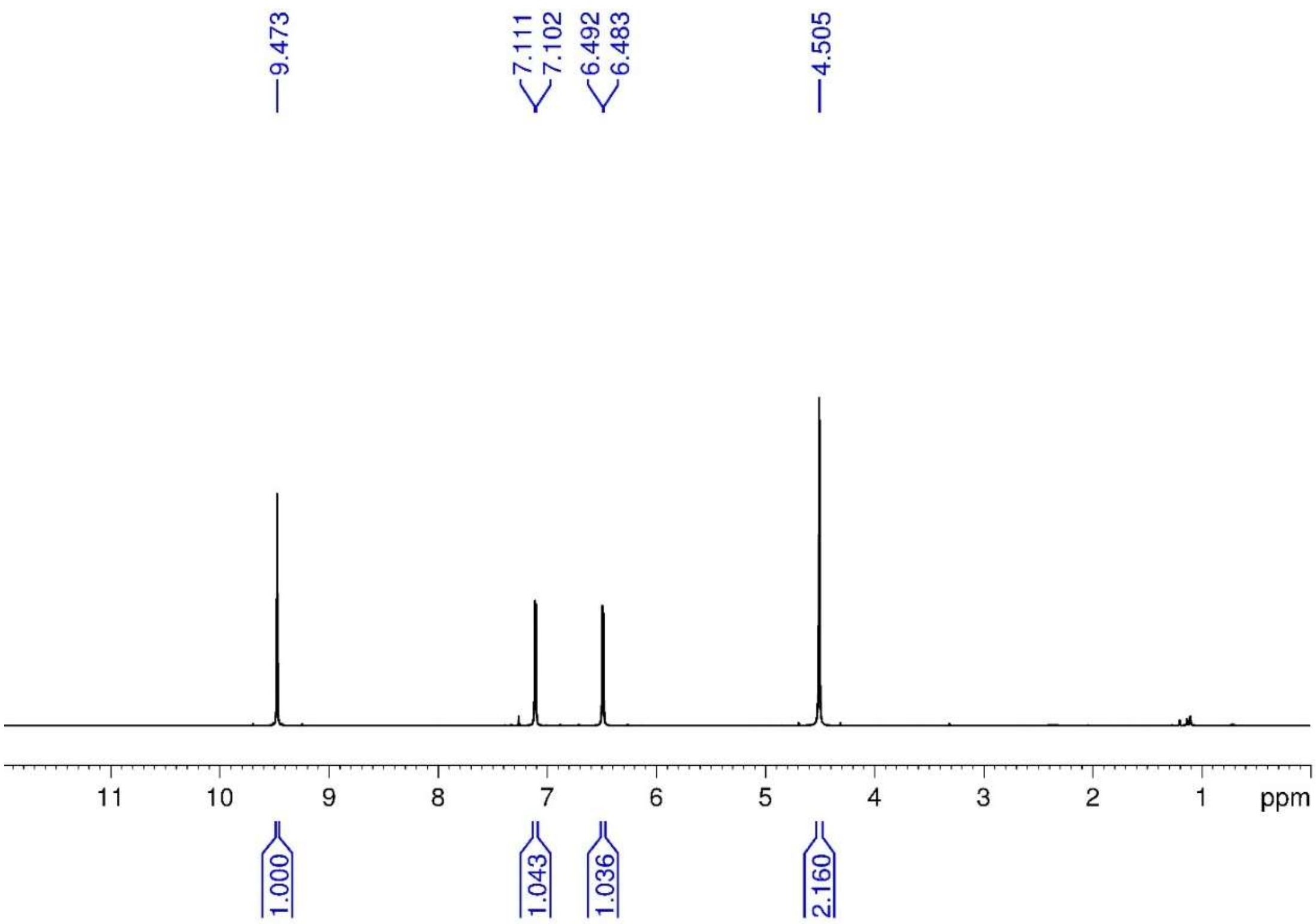

Figure S2. The ^{1}H-NMR spectrum of **CMF**.

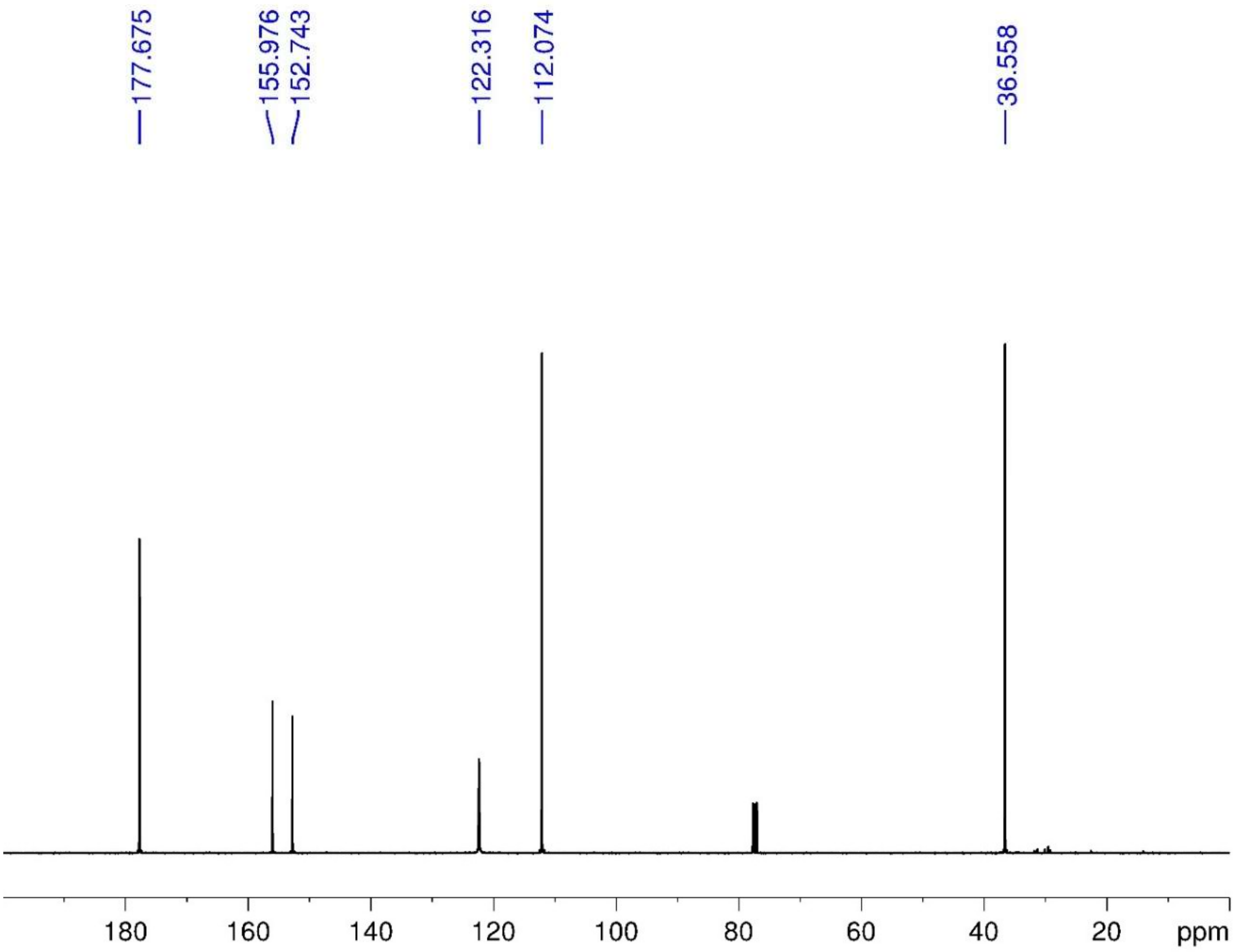

Figure S3. The ^{13}C-NMR spectrum of **CMF**.

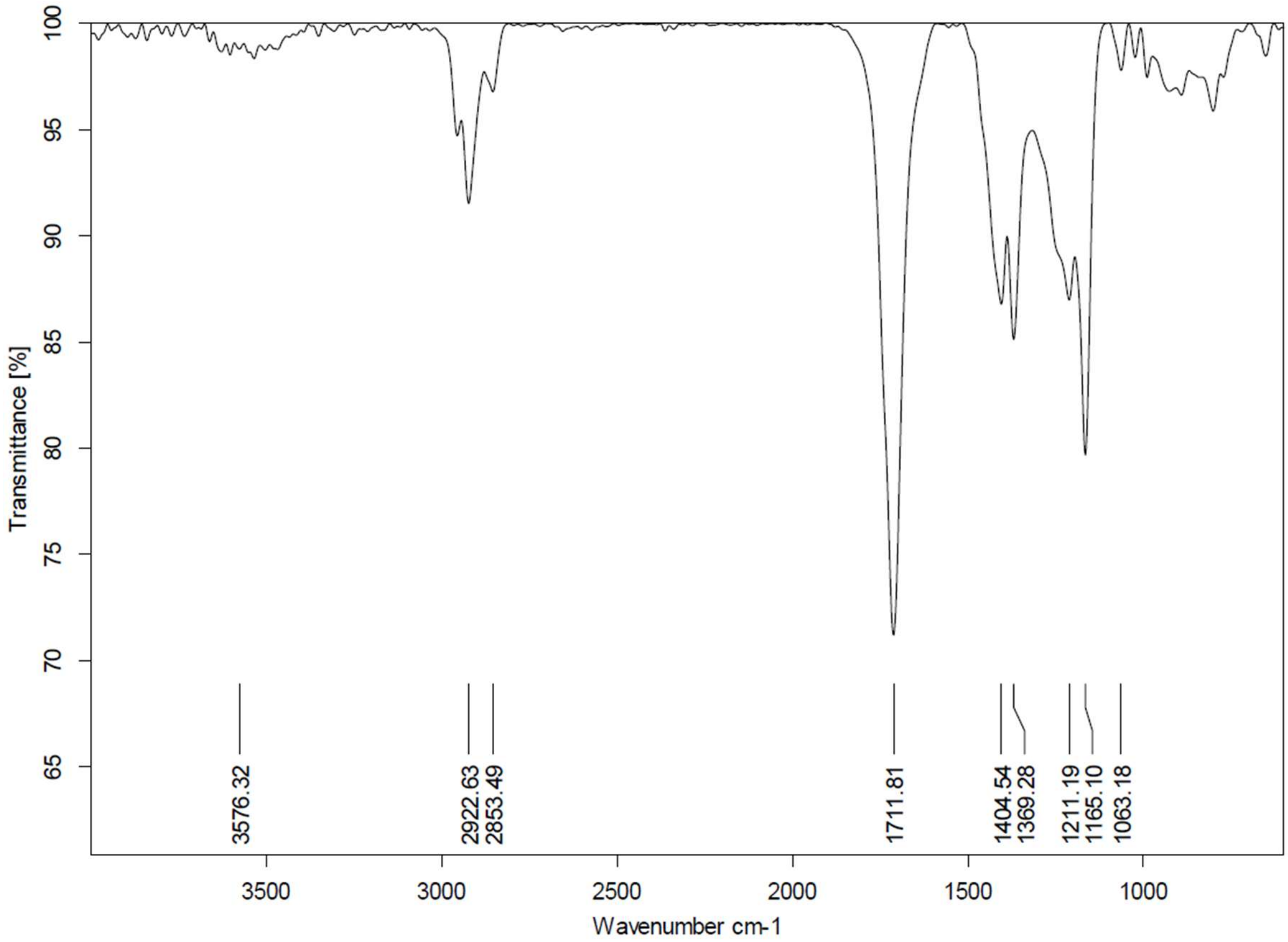

Figure S4. The FTIR spectrum of **LA**.

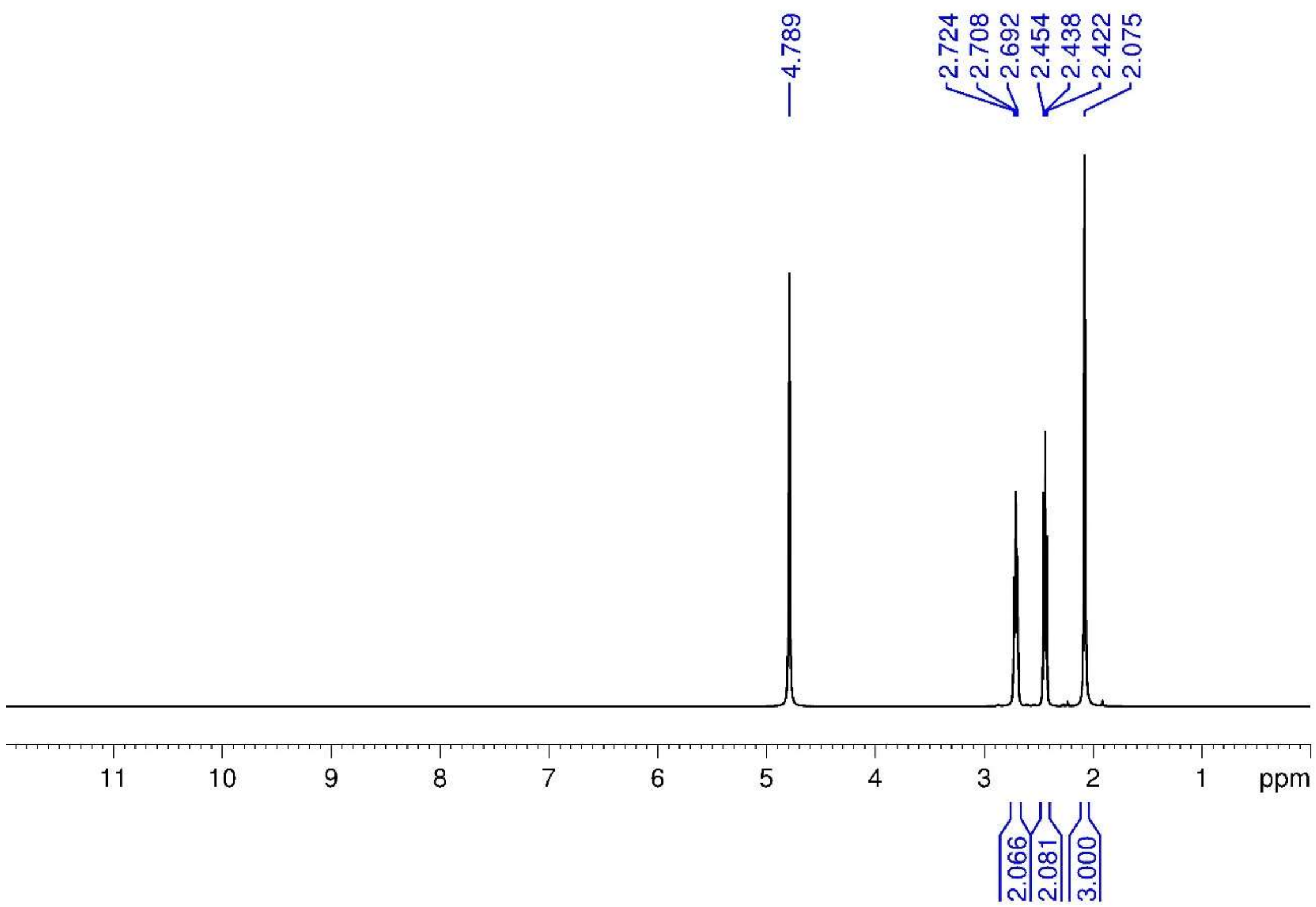

Figure S5. The ^{1}H-NMR spectrum of **LA**.

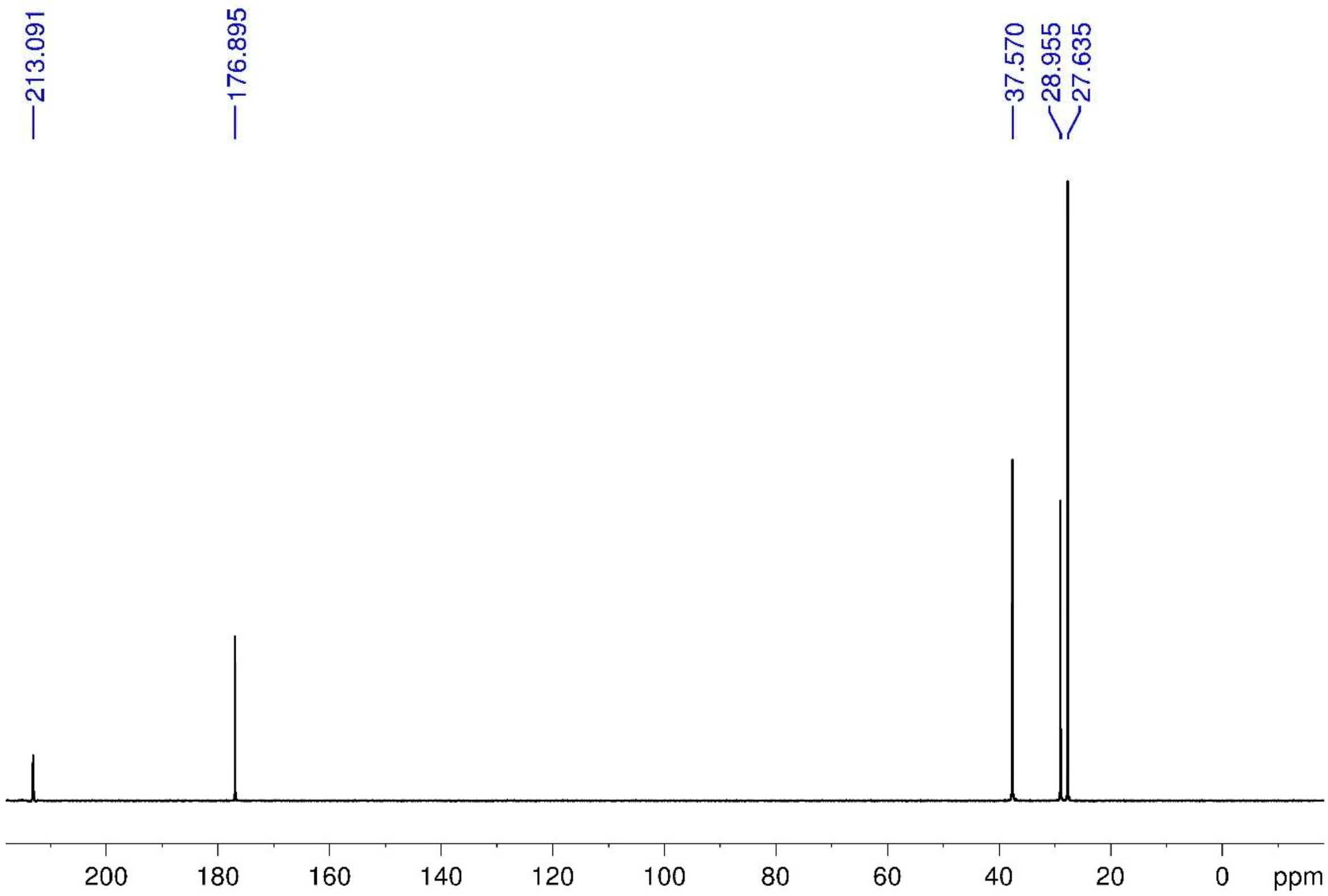

Figure S6. The ^{13}C-NMR spectrum of **LA**.

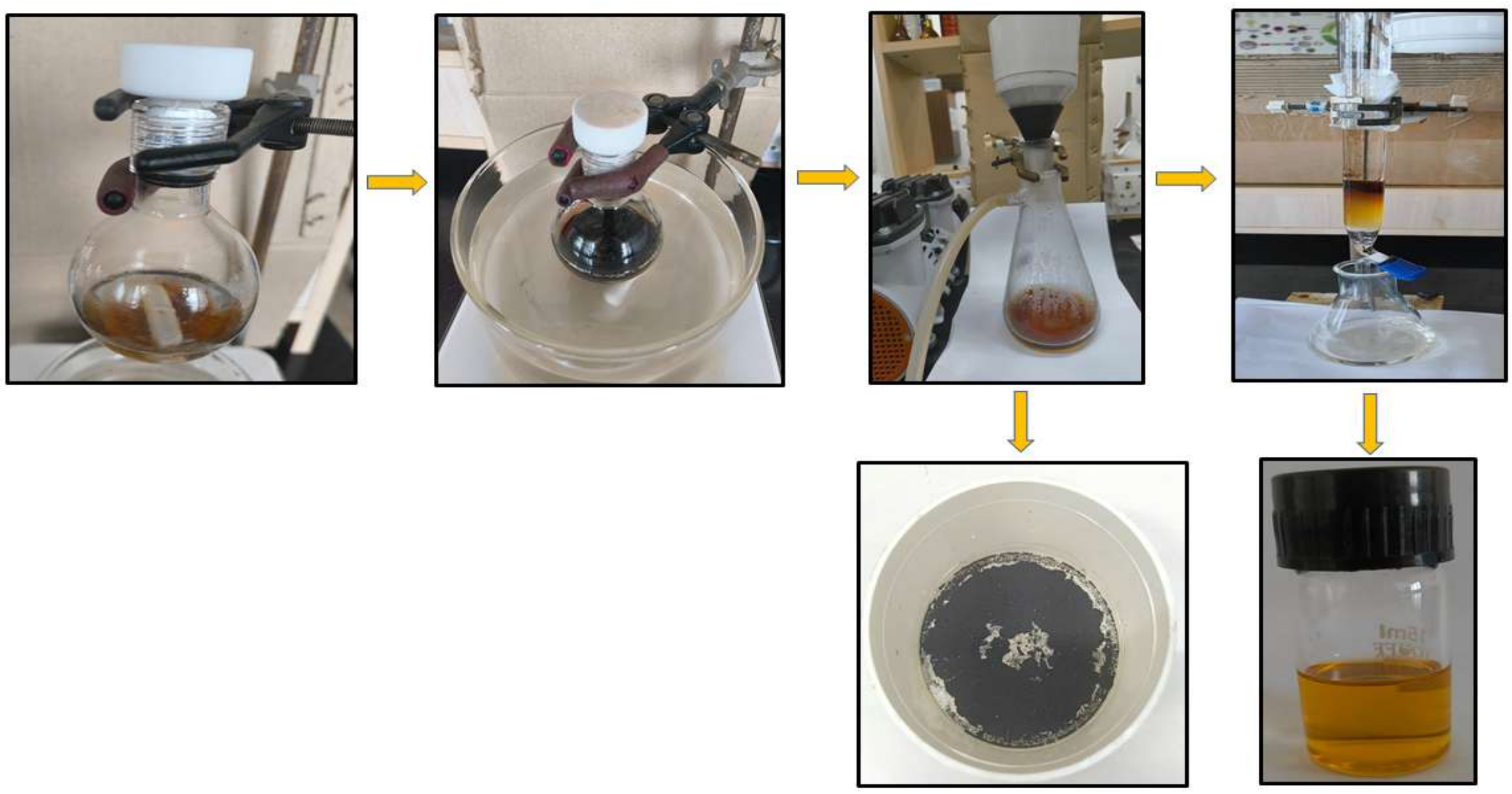

Figure S7. Reaction setup, workup, and purification of **LA** under conventional heating.

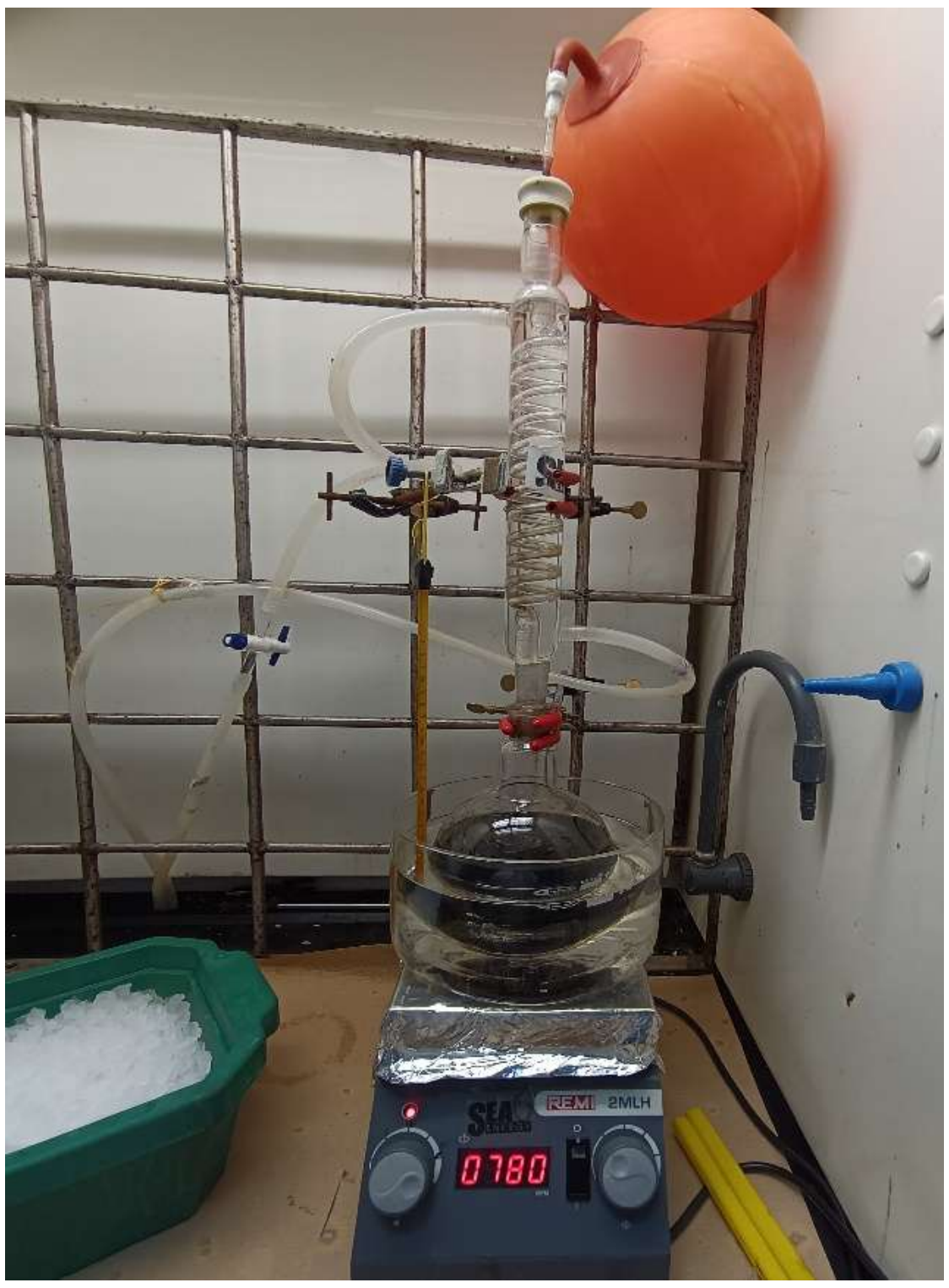

Figure S8. Scale-up synthesis of **LA** from *Spinosum* dried seaweed flakes.

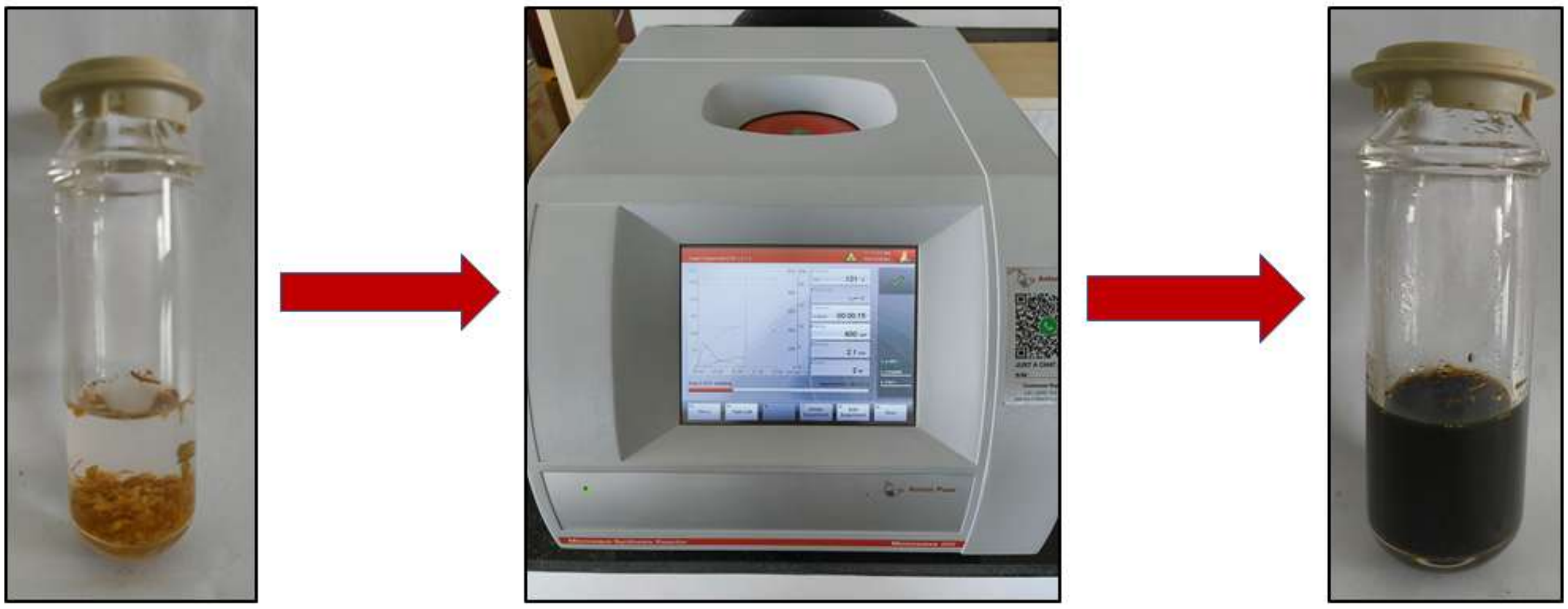

Figure S9. Reaction mixture before and after microwave-assisted **LA** synthesis.

Steps

Step	Program	Temperature	Time	Power	Cooling	Stirrer Speed
		°C	hh:mm:ss	W		rpm
1	Heat as fast as possible	80	-	850	Off	600
2	Hold	-	00:30:00	850	Off	600
3	Cool down	55	-	-	On	600

Experiment Result

► **Result:** OK

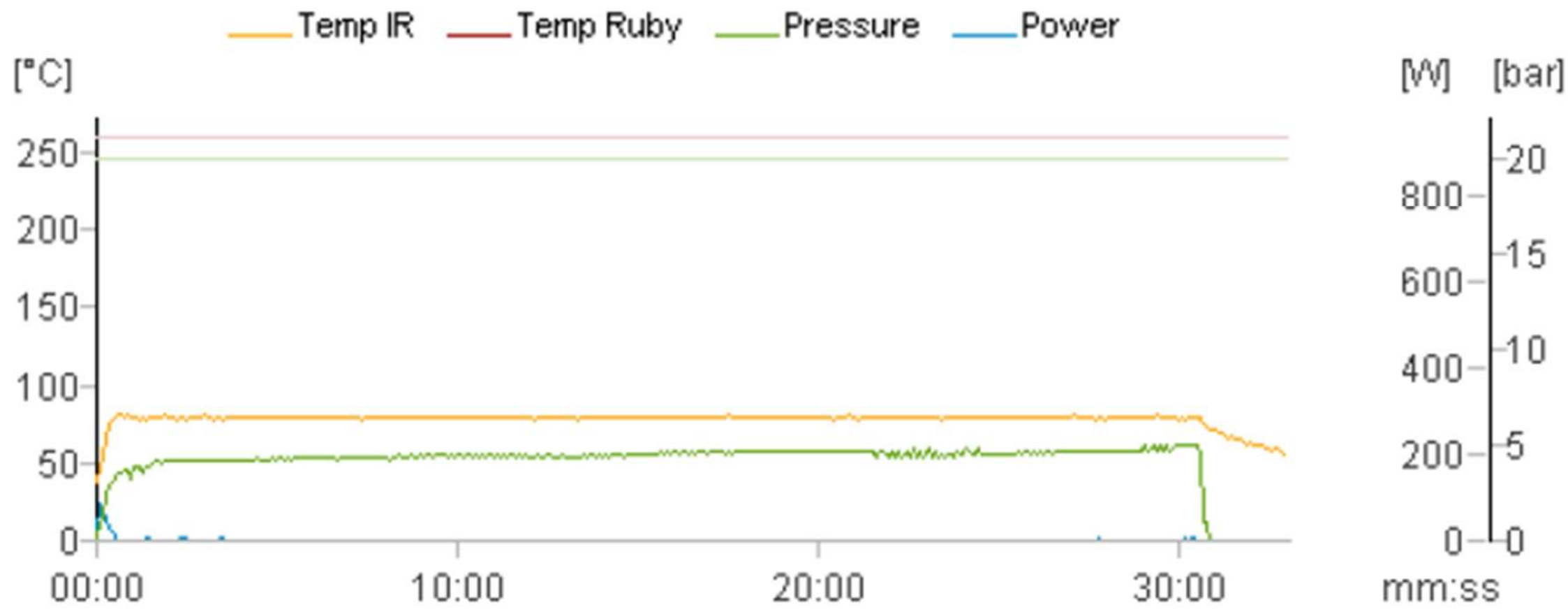

Figure S10. Reaction profile diagram for the microwave-assisted synthesis of **CMF** from *Spinosum* dried seaweed flakes.

Steps

Step	Program	Temperature	Time	Power	Cooling	Stirrer Speed
		°C	hh:mm:ss	W		rpm
1	Heat as fast as possible	120	-	850	Off	600
2	Hold	-	00:30:00	850	Off	600
3	Cool down	55	-	-	On	600

Experiment Result

► **Result:** OK

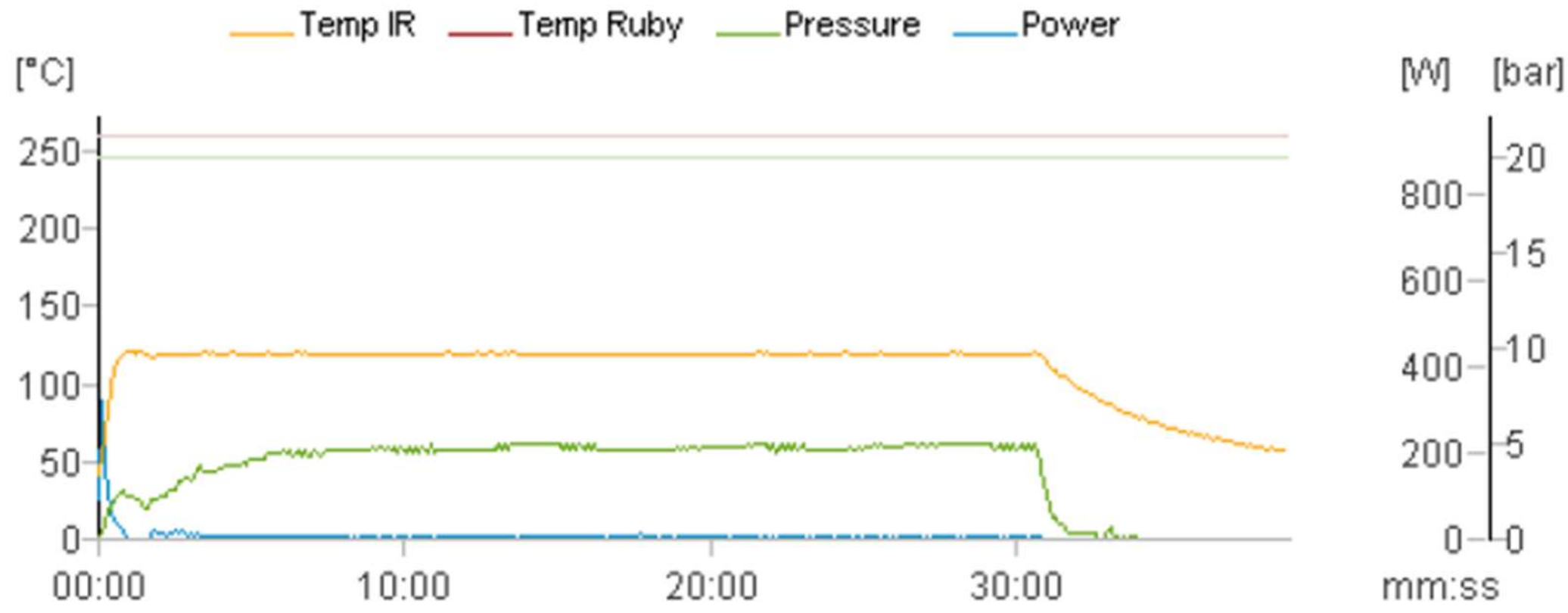

Figure S11. Reaction profile diagram for the microwave-assisted synthesis of **LA** from *Spinosum* dried seaweed flakes.

Table S1. Molar yields of LA obtained from DSF under different reaction conditions.

Entry	Reaction condition	Yield (mol%) #
1	1 N HCl, 120 °C, 4 h	24
2	3 N HCl, 120 °C, 4 h	30
3	6 N HCl, 120 °C, 4 h	43
4	9 N HCl, 120 °C, 4 h	31
5	6 N HCl, 100 °C, 4 h	22
6	6 N HCl, 110 °C, 4 h	27
7	6 N HCl, 130 °C, 4 h	39

8	6 N HCl, 140 °C, 4 h	35
9	6 N HCl, 100 °C, 2 h	25
10	6 N HCl, 100 °C, 6 h	39
11	6 N HCl, 100 °C, 8 h	34
12	TEAHS, 120 °C, 4 h	10
13	PTA, 120 °C, 4 h	16
14	NR50, 120 °C, 4 h	14
15	6 N HCl, TEAHS, 120 °C, 4 h	53
16	6 N HCl, Potash alum, 120 °C, 4 h	33
17	6 N HCl, $CrCl_2$, 120 °C, 4 h	45
18	6 N HCl, $AlCl_3$, 120 °C, 4 h	51
19	6 N HCl, $SnCl_4$, 120 °C, 4 h	53
20	6 N HCl, formic acid, 120 °C, 4 h	57

# indicates that the molar yields were calculated by assuming 70 wt% of ι-carrageenan is present in DSF based on literature data. The moisture content of 16 wt% was considered in calculating the carrageenan content on a dry-weight basis.

LA yield (mol%) = [(2 × moles of LA obtained) / (moles of ι-carrageenan)] × 100

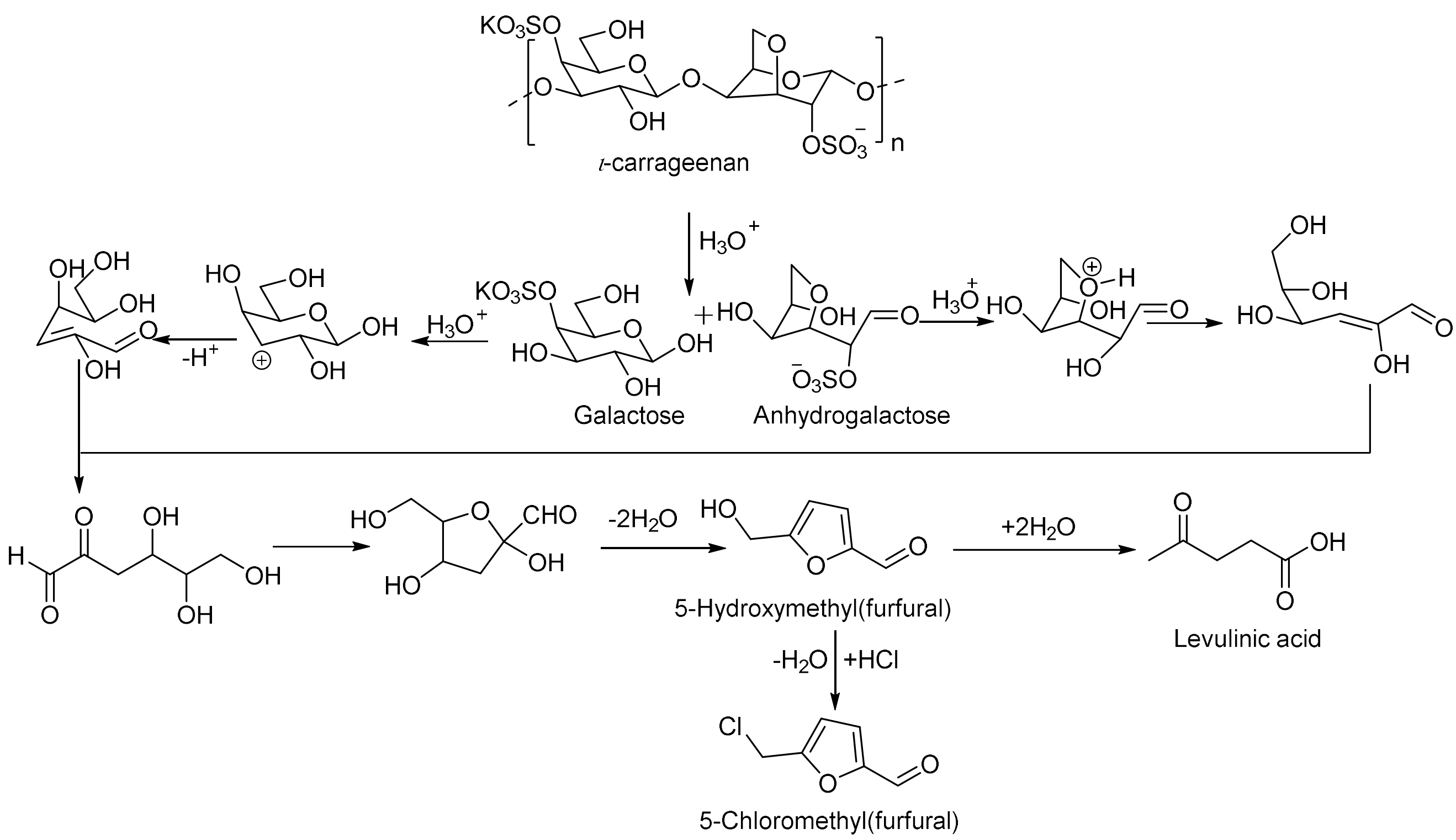

Scheme S1. Reaction mechanism for the synthesis of LA and CMF from ι-carrageenan (adapted from Ref. 1).

References

1. P. Naik C, A. K. Yadav, D. A. Aranha and S. Dutta, *Biomass Convers. Biorefin.*, DOI:10.1007/s13399-024-06484-3.
